# Circulating protein biomarkers and their association with vulnerable plaque characteristics – a PROSPECT II substudy

**DOI:** 10.1016/j.ijcrp.2025.200440

**Published:** 2025-05-24

**Authors:** Tania Sharma, Akiko Maehara, Michael Maeng, Lars Kjøller-Hansen, Thomas Engstrøm, Ori Ben-Yehuda, Mitsuaki Matsumura, Ole Fröbert, Jonas Persson, Rune Wiseth, Alf Inge Larsen, Sasha Koul, Rebecca Rylance, Gary S. Mintz, Ziad A. Ali, Stefan K. James, Gregg W. Stone, David Erlinge

**Affiliations:** a Department of Cardiology, Clinical Sciences, Lund University, Lund, Sweden; b New York-Presbyterian Hospital and Columbia University Irving Medical Center, New York, NY, USA; c Cardiovascular Research Foundation, New York, NY, USA; d Department of Cardiology, Aarhus University Hospital, and Department of Clinical Medicine, Aarhus University, Aarhus, Denmark; e Zealand University Hospital, Roskilde, Denmark; f Rigshospitalet, University of Copenhagen, Copenhagen, Denmark; g University of California San Diego, San Diego, CA, USA; h Orebro University, Faculty of Health, Orebro, Sweden and Department of Biomedicine, Aarhus University, Denmark; i Department of Clinical Sciences, Danderyd University Hospital, Karolinska Institutet, Stockholm, Sweden; j St Olavs Hospital, Trondheim, Norway; k Stavanger University Hospital, Stavanger, Norway; l Department of Medical Sciences and Uppsala Clinical Research Center, Uppsala University, Uppsala, Sweden; m The Zena and Michael A. Wiener Cardiovascular Institute, Icahn School of Medicine at Mount Sinai, New York City, NY, USA

**Keywords:** Vulnerable plaque, Myocardial infarction, Plaque burden, Lipid rich plaque, Atherosclerosis

## Abstract

**Background:**

In the PROSPECT-II study, near infrared spectroscopy (NIRS) and intravascular ultrasound (IVUS) was used to characterize atherosclerotic plaques in the coronary arteries. NIRS-derived lipid core burden index (LCBI) and IVUS-derived plaque burden (PB) were able to identify plaques strongly associated with adverse cardiovascular events.

**Aim:**

Our aim was to identify biomarkers associated with LCBI or PB in the coronary arteries.

**Methods:**

898 patients with recent myocardial infarction underwent percutaneous coronary intervention. Blood samples to analyze plasma levels of 179 proteins associated with cardiovascular disease were procured and a combined NIRS-IVUS catheter was used to analyze the coronary arteries. Adjusted linear regression models were calculated between the biomarkers and the outcomes of interest, adjusted for multiplicity testing. Kaplan-Meier survival curves of biomarkers divided by median were assessed with the log-rank test. Adjusted Cox proportional models were calculated for major adverse cardiovascular events.

**Results:**

A total of 24 proteins were associated with PB and 28 proteins with LCBI. Eight of these biomarkers were associated with both increased pan-coronary LCBI and PB; IL-18R1, CSF-1, VEGFA, EN-RAGE, cathepsin D, PCSK9, transferrin receptor protein 1 and OPN. After adjusting for multiplicity, angiopoietin like 3 (ANGPTL3) retained its association with LCBI, and IL-18R1 and CSF-1 retained their association with PB.

**Conclusion:**

We were able to identify distinct biomarker patterns associated with PB and LCBI. IL-18R1 and CSF-1 had a strong relationship with PB. ANGPTL3 was associated with lipid rich plaques but not with PB, supporting its role in lipid accumulation and development of vulnerable plaques.

## Highlights

• This is a Prospective study conducted across 16 centers in Sweden, Norway and Denmark.
• 898 patients with myocardial infarction had 3692 lesions analyzed by near infrared spectroscopy (NIRS) and intravascular ultrasound (IVUS).
• 179 protein biomarkers associated with cardiovascular disease were assessed using Proximity Extension Assay proteomics.
• IL-18R1 and CSF-1 were linked to Plaque burden. ANGPTL3 was associated with lipid rich plaques.
• This is the first study to combine plaque burden and lipid core burden index with biobanking in coronary arteries.

## Abbreviations

ACS =: acute coronary syndrome
FDR =: false discovery rate
HDL =: high density lipoprotein
IVUS =: intravascular ultrasound
LDL =: low density lipoprotein
LCBI =: lipid core burden index
MACE =: major adverse cardiac events
MaxLCBI_4mm_ =: maximum LCBI within any 4 mm segment across the entire lesion
NIRS =: near infrared spectroscopy
PB =: plaque burden
PCI =: percutaneous coronary intervention
PEA =: proximity extension assay
ANGPTL3 =: Angiopoietin-related protein 3
AZU1 =: Azurocidin
BNP =: Natriuretic peptides B
CCL16 =: C-C motif chemokine 16
CCL19 =: C-C motif chemokine 19
CCL23 =: C-C motif chemokine 23
CDH5 =: Cadherin-5
CD163 =: Scavenger receptor cysteine-rich type 1 protein
CHI3L1 =: Chitinase-3-like protein 1
CHIT1 =: Chitotriosidase-1
CHL1 =: Neural cell adhesion molecule L1-like protein
CSF-1 =: Macrophage colony-stimulating factor 1
CTSD =: Cathepsin D
CTSZ =: Cathepsin Z
EN-RAGE =: Protein S100-A12
Gal-9 =: Galectin-9
HO-1 =: Heme oxygenase 1
IGFBP-2 =: Insulin-like growth factor-binding protein 2
IL-10RB =: Interleukin-10 receptor subunit beta
IL-12B =: Interleukin-12 subunit beta
IL-17RA =: Interleukin-17 receptor A
IL-18R1 =: Interleukin-18 receptor 1
IL-1ra =: Interleukin-1 receptor antagonist protein
IL-6 =: Interleukin-6
ITGB2 =: Integrin beta-2
KIT =: Mast/stem cell growth factor receptor Kit
LAP TGF-beta-1 =: Latency-associated peptide transforming growth factor beta-1
LPL =: Lipoprotein lipase
LTBR =: Lymphotoxin-beta receptor
MB =: Myoglobin
MCP-3 =: Monocyte chemotactic protein 3
MMP-9 =: Matrix metalloproteinase-9
MMP12 =: Matrix metalloproteinase-12
MPO =: Myeloperoxidase
NOTCH3 =: Neurogenic locus notch homolog protein 3
OPN =: Osteopontin
OSM =: Oncostatin-M
PCSK9 =: Proprotein convertase subtilisin/kexin type 9
PD-L1 =: Programmed cell death 1 ligand 1
PGF =: Placenta growth factor
PON3 =: Paraoxonase
PRSS2 =: Trypsin-2
PRTN3 =: Myeloblastin
RAGE =: Receptor for advanced glycosylation end products
SPON1 =: Spondin-1
ST2 =: ST2 protein
ST6GAL1 =: Beta-galactoside alpha-2,6-sialyltransferase 1
TIMP1 =: Metalloproteinase inhibitor 1
TNC =: Tenascin
TR =: Transferrin receptor protein 1
TWEAK =: Tumor necrosis factor (Ligand) superfamily, member 12
VASN =: Vasorin
VEGFA =: Vascular endothelial growth factor A

## 1 Introduction

Cardiovascular disease continues to be the leading cause of mortality worldwide [1] with rupture of a vulnerable plaque precipitating the majority of acute coronary events [2]. Intravascular imaging has prospectively demonstrated that high-risk vulnerable plaques are characterized by a thin fibrous cap overlying a large lipid core together with active inflammation [3]. Intravascular ultrasound (IVUS), near-infrared spectroscopy (NIRS) and optical coherence tomography (OCT) are intravascular imaging techniques that have been validated in identifying these vulnerable atherosclerotic plaques [[4], [5], [6], [7], [8], [9]].

IVUS characterizes the lumen area and vessel wall in vivo and has the ability to provide quantitative measurements and detailed information about coronary plaque volume [10]. The Providing Regional Observations to Study Predictors of Events in the Coronary Tree (PROSPECT) study was the first to show that IVUS-derived plaque characteristics, especially plaque burden (PB) are independently associated with major adverse cardiovascular events (MACE) arising from untreated “non-culprit” lesions during follow-up in patients with acute coronary syndromes (ACS) [8].

NIRS, a catheter-based spectroscopic modality, can identify lipid rich cores in atherosclerotic plaques, quantified as the lipid core burden index (LCBI) [4]. NIRS-derived plaque outcomes have also been associated with MACE in patients with coronary artery disease, with a marked increase in risk over several years of follow-up [[11], [12], [13]]. These studies have demonstrated that NIRS-IVUS derived variables are effective in identifying high-risk coronary plaques in vivo, providing prognostic value for future cardiovascular events. Large prospective trials such as the Lipid Rich Plaque (LRP) and the Providing Regional Observations to Study Predictors of Events in the Coronary Tree II (PROSPECT II) studies [5,7] have shown that the presence of high LCBI and high PB is a hallmark of vulnerable, non-flow limiting plaques that may cause major adverse cardiovascular events.

Identification of circulating biomarkers associated with the presence of vulnerable plaque characteristics could enable a non-invasive strategy to improve early diagnosis and risk stratification. However, few biomarker studies are available with information of both lipid core and PB.

The aim of the present study was to evaluate and identify biomarkers associated with NIRS-IVUS related vulnerable plaque characteristics as expressed by LCBI and PB. We sought to find candidate proteins to further elucidate the mechanisms of the development of vulnerable plaques.

## 2 Method

### 2.1 Study population

The PROSPECT II study is a prospective study conducted at 16 centers in Sweden, Denmark and Norway between 2014 and 2017. Nine hundred and two patients with recent myocardial infarction (MI) were enrolled after successful Percutaneous coronary intervention (PCI) of all flow-limiting culprit lesions. A combined NIRS-IVUS catheter was used to identify all non-culprit lesions in the proximal 6–10 cm of all three coronary arteries. Non-culprit lesions were defined as untreated coronary segments ≥2 mm in length with ≥40 % PB. The absence of non-culprit lesions led to the de-enrollment of 4 patients, resulting in a cohort of 898 patients where a total of 3629 untreated non-culprit lesions were prospectively characterized. Blood samples for further analysis could not be obtained from 21 patients, bringing the final population count to 877. Clinical follow up occurred at 1, 6, 12 months and annually thereafter. Detailed inclusion and exclusion criteria are listed in Supplementary appendix Table S1. This study was conducted in accordance with the criteria described in the Declaration of Helsinki and was approved by the ethics committee in each country. All patients signed informed consent before enrollment.

### 2.2 Biobank and biomarkers

Prior to PCI, blood samples were drawn, centrifuged within 2 h, and then stored at a temperature of −80 °C. Biomarkers were assessed using Proximity Extension Assay (PEA) technology by Olink Proteomics at the Clinical Biomarkers Facility, Science for Life Laboratory, Uppsala University. The analyzing staff were blinded to the NIRS-IVUS data. In this study we chose to analyze 179 biomarkers from four PEA panels: Cardiometabolic, Cardiovascular II, Cardiovascular and Inflammation. These panels have previously been associated with cardiovascular disease. See Supplementary Appendix Table S2 and S3.

PEA is a technique which enables the simultaneous analysis of large amounts of protein biomarkers in small volumes of plasma. The high level of multiplexing is achieved through oligonucleotide labelled antibody pairs binding to their target proteins in each sample and forming a PCR reporter sequence. A signal is only produced when the correct antibody pairs are matched. This sequence is then amplified, detected, and quantified, resulting in high sensitivity for low volumes [14,15]. The generated data is expressed as an arbitrary unit, normalized protein eXpression, presented on a log2scale.

### 2.3 Imaging analysis

Prospective analysis of the intravascular images was conducted at the Cardiovascular Research Foundation, New York, NY, USA without prior knowledge of patient outcomes [7]. Using an automated edge-detection algorithm (QAngio XA 7.3, Medis Medical Imaging Systems, Leiden, Netherlands), angiographic analyses were performed on all lesions with ≥30 % diameter stenosis in each epicardial vessel and side branches with a diameter ≥1.5 mm.

The spectroscopic data from NIRS generates a chemogram, a color-coded distribution of lipid probability with the y-axis corresponding to the circumferential position (1°/pixel) and the x-axis corresponding to the axial vessel position (0.1 mm/pixel). The LCBI is the fraction of pixels with a probability of lipid >0.6 divided by all pixels within the region of interest, multiplied by 1000. Pan-coronary LCBI was defined as the total untreated lipid core burden index per patient [6].

IVUS evaluation of all image cross-sections and quantitative measurements were performed using CASS IntraVascular 2.1 software (Pie Medical Imaging, Maastricht, Netherlands). Pan-coronary plaque burden was defined as vessel total plaque volume/total vessel volume.

### 2.4 Outcomes and definitions

The primary imaging endpoints measured were pan-coronary LCBI and pan-coronary PB. They represented the average lipid content and PB in the three coronary arteries. The secondary endpoint of MACE was the composite rate of cardiac death, MI or unstable or progressive angina either requiring hospitalization or revascularization with rapid lesion progression.

### 2.5 Statistical analysis

The PEA technique can reveal an antigen excess relative to the reagent antibodies. This is called the high-dose hook effect and results in falsely low values [16]. We identified these biomarkers from the study sample; violin plots were graphed to exclude proteins demonstrating a hook effect. Biomarkers and NIRS-IVUS measurements (all continuous variables) were examined for normal distribution by visual inspection of histograms.

Linear regression models adjusted for age and sex were calculated between 179 biomarkers and the imaging outcomes. A false discovery rate (FDR) correction was performed to control for multiplicity and a q-value <0.05 was deemed significant.

Cox proportional hazards models adjusted for age and sex were used to analyze the association between biomarker concentration divided into two groups by the median and MACE. These results were presented as hazard ratios with 95 % confidence intervals. Kaplan-Meier curves were presented with log-rank tests.

All tests were two-sided, and all analyses were performed in Stata (version SE 17.0).

## 3 Results

### 3.1 Baseline characteristics

Baseline clinical and imaging characteristics are summarized in Table 1. Median age was 63 years, 83 % were men, and 12.1 % had diabetes mellitus. Baseline characteristic comparisons between the low and high groups for plaque burden (PB) and lipid core burden index (LCBI) are presented in the Supplementary Tables S2 and S3.

**Table 1:** Baseline characteristics.

Parameter	n = 877
Age (years)	63.0 (55.0, 70.0)
Sex, female	17.2 % (151/877)
Current or recent smoker	31.9 % (276/865)
Ex-smoker	31.6 % (273/865)
Never smoked	36.5 % (316/865)
Diabetes mellitus, all	12.1 % (106/877)
Insulin-treated	4.3 % (38/877)
Prior percutaneous coronary intervention	12.1 % (106/877)
Hypertension requiring medication	37.3 % (327/877)
Hyperlipidemia requiring medication	25.2 % (221/877)
Clinical presentation	
ST-segment elevation myocardial infarction	22.2 % (195/877)
Non-ST-segment elevation myocardial infarction	77.8 % (682/877)

**IVUS measurements**	

Number of IVUS non-culprit lesions∗∗	4.0 (3.0, 5.0)
Number of diseased vessels (with ≥1 IVUS non-culprit lesion)	2.0 (2.0, 3.0)
≥1 lesions with plaque burden ≥70 %	59.1 % (518/877)
Total percent plaque volume (%)	42.5 (37.9, 46.8)
**NIRS measurements**	
Total Untreated Coronary Artery LCBI per patient	50.1 (25.0, 79.5)
≥1 lesions with MaxLCBI4mm ≥ Upper quartile (324.7)	58.6 % (506/863)

Continuous data are median (Q1, Q3). ∗∗Untreated coronary segments ≥2 mm in length with ≥40 % plaque burden. IVUS denotes intravascular ultrasound; NIRS, near infrared spectroscopy; LCBI, lipid core burden index.

### 3.2 Association between plaque burden and biomarkers

**Fig. 1:**
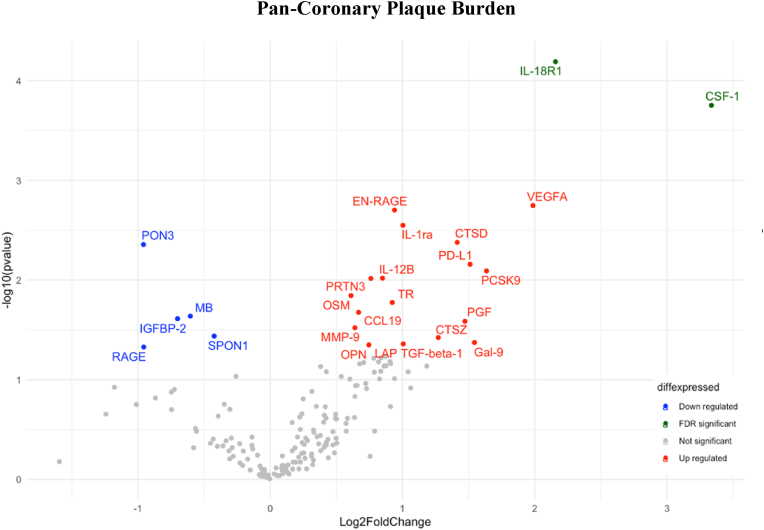
Pan-Coronary Plaque Burden (PB) Volcano plot showing the association between plaque burden (PB) and 179 circulating biomarkers associated with cardiovascular disease. The horizontal axis is the log2 fold change between biomarkers and PB. The negative log10 of the P-value of Fisher's exact test is plotted on the vertical axis. Each biomarker is represented by one point on the graph. Significantly upregulated biomarkers are represented in red; significantly downregulated biomarkers are represented in blue; the biomarkers which retained a significant relationship after adjusted false rate discovery rate correction are represented in green.

In total, 24 proteins showed an association (unadjusted p < 0.05) with pan-coronary PB using linear regression (Table 2). Some of the proteins with associations were interleukin-18 receptor 1 (IL-18R1), colony stimulating factor 1 (CSF-1), proprotein convertase subtilisin/kexin type 9 (PCSK9), matrix metalloproteinase-9 (MMP-9), and vascular endothelial growth factor A (VEGFA). After correction with FDR, IL-18R1 (q = 0.012) and CSF-1 (q = 0.016) retained their statistical significance (Fig. 1).

**Table 2:** Association between biomarkers and NIRS-IVUS related outcomes.

Pan-coronary Lipid Core Burden Index (LCBI)		Pan-coronary Plaque Burden (PB)		Biomarkers associated with both LCBI and PB
**Biomarker**	**P value**	**Biomarker**	**P value**	**Biomarker**
CHI3L1	0.000	IL-18R1∗	6,430E-05	IL-18R1∗
TR	0.001	CSF-1∗	0.000	CSF-1∗
ANGPTL3∗	0.001	VEGFA	0.002	VEGFA
TIMP1	0.002	EN-RAGE	0.002	PCSK9
CCL23	0.002	IL-1ra	0.003	EN-RAGE
VEGFA	0.003	CTSD	0.004	CTSD
MCP-3	0.003	PON3	0.004	TR
BNP	0.006	PD-L1	0.007	OPN
TNC	0.006	PCSK9	0.008	
PRSS2	0.007	IL-12B	0.010	
ST6GAL1	0.010	PRTN3	0.010	
MPO	0.011	OSM	0.014	
IL-6	0.011	TR	0.017	
ST2	0.014	CCL19	0.021	
EN-RAGE	0.015	MB	0.023	
CTSD	0.016	IGFBP-2	0.024	
OPN	0.017	PGF	0.026	
LPL	0.019	MMP-9	0.030	
AZU1	0.019	SPON1	0.037	
CHIT1	0.020	CTSZ	0.038	
HO-1	0.020	Gal-9	0.042	
CHL1	0.021	LAP TGF-beta-1	0.044	
IL-18R1	0.022	OPN	0.045	
TWEAK	0.028	RAGE	0.047	
PCSK9	0.031			
CDH5	0.034			
MMP12	0.046			
CSF-1	0.049			

∗Significant after false discovery rate correction (q value < 0.05). Denotations of the protein abbreviations; CHI3L1; Chitinase-3-like protein 1, TR; Transferrin receptor protein 1, ANGPTL3; Angiopoietin-related protein 3, TIMP1; Metalloproteinase inhibitor 1, CCL23; C-C motif chemokine 23, VEGFA; Vascular endothelial growth factor A, MCP-3; Monocyte chemotactic protein 3, BNP; Natriuretic peptides B, TNC; Tenascin, PRSS2; Trypsin-2, ST6GAL1; Beta-galactoside alpha-2,6-sialyltransferase 1, MPO; Myeloperoxidase, IL-6; Interleukin-6, ST2; ST2 protein, EN-RAGE; Protein S100-A12, CTSD; Cathepsin D, OPN; Osteopontin, LPL; Lipoprotein lipase, AZU1; Azurocidin, CHIT1; Chitotriosidase-1, HO-1; Heme oxygenase 1, CHL1; Neural cell adhesion molecule L1-like protein, IL-18R1; Interleukin-18 receptor 1, TWEAK; Tumor necrosis factor (Ligand) superfamily, member 12, PCSK9; Proprotein convertase subtilisin/kexin type 9, CDH5; Cadherin-5, MMP12; Matrix metalloproteinase-12, CSF-1; Macrophage colony-stimulating factor 1, IL-1ra; Interleukin-1 receptor antagonist protein, PON3; Paraoxonase, PD-L1; Programmed cell death 1 ligand 1, IL-12B; Interleukin-12 subunit beta, PRTN3; Myeloblastin, OSM; Oncostatin-M, CCL19; C-C motif chemokine 19, MB; Myoglobin, IGFBP2; Insulin-like growth factor-binding protein 2, PGF; Placenta growth factor, MMP-9; Matrix metalloproteinase-9, SPON1; Spondin-1, CTSZ; Cathepsin Z, Gal9; Galectin-9, LAP TGF-beta-1; Latency-associated peptide transforming growth factor beta-1, RAGE; Receptor for advanced glycosylation end products.

### 3.3 Association between LCBI burden and biomarkers

**Fig. 2:**
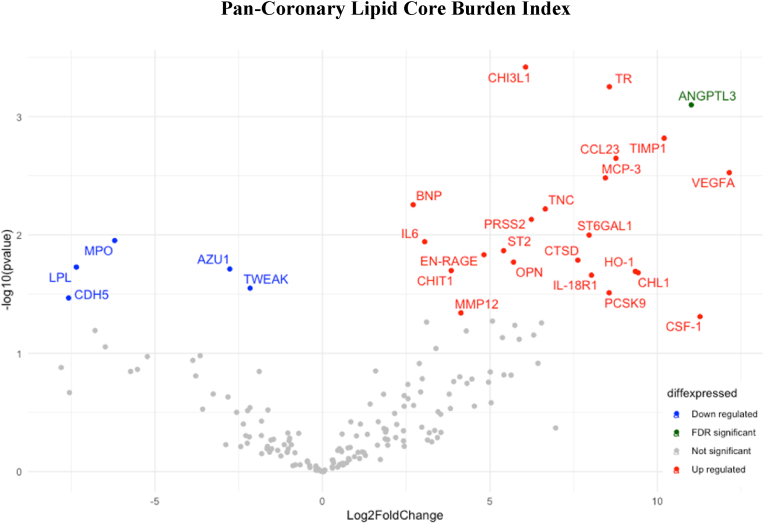
Pan-Coronary Lipid Core Burden Index (LCBI) Volcano plot showing the association between lipid core burden index (LCBI) and 179 circulating biomarkers associated with cardiovascular disease. The horizontal axis is the log2 fold change between biomarkers and plaque burden (PB). The negative log10 of the P-value of Fisher's exact test is plotted on the vertical axis. Each biomarker is represented by one point on the graph. Significantly upregulated biomarkers are represented in red; significantly downregulated biomarkers are represented in blue; the biomarkers which retained a significant relationship after adjusted false rate discovery rate correction are represented in green.

Using linear regression, an association (unadjusted p < 0.05) was found between 28 proteins and pan-coronary LCBI. Eight of these proteins also demonstrated an association with pan-coronary PB, as shown in Table 2. Proteins positively associated with LCBI included chitinase 3 like protein 1 (CHI3L1), angiopoietin-like 3 (ANGPTL3), interleukin 6 (IL-6), lipoprotein lipase (LPL), PCSK9, IL-18R1 and CSF-1. After FDR correction, ANGPTL3 (q = 0.048) was the only biomarker to retain a statistically significant relationship with LCBI (Fig. 2).

### 3.4 Association between other morphological characteristics and biomarkers

The protein biomarkers were also analyzed with linear regression for their relationship with the worst maxLCBI_4mm_, presence of ≥1 lesion with maxLCBI_4mm_ ≥ upper quartile (324.7), worst PB, maxPB ≥70 %, or combinations thereof. We identified several proteins with an unadjusted association with p < 0.05. However, none retained significance after FDR (results not shown).

### 3.5 MACE

The median follow-up period was 3.6 (IQR 2.6–4.0) years, during which 7,3 % of patients experienced a non-culprit lesion related MACE. In the adjusted models, C-C motif chemokine 19 (CCL19), Scavenger receptor cysteine-rich type 1 protein (CD163), CSF-1, CTSZ, Interleukin-10 receptor subunit beta (IL-10RB), Interleukin-12 subunit beta (IL-12B), Interleukin-17 receptor A (IL-17RA), Integrin beta-2 (ITGB2), stem cell growth factor receptor Kit (KIT), Lymphotoxin-beta receptor (LTBR), Neurogenic locus notch homolog protein 3 (NOTCH3), Programmed cell death 1 ligand 1 (PD-L1), Myeloblastin (PRTN3), Metalloproteinase inhibitor 1 (TIMP1) and Vasorin (VASN) were significantly associated with non-culprit lesion related MACE. Kaplan-Meier survival curves and a forest plot illustrating the associations for these biomarkers are presented in the Supplementary Appendix (Figure S1 and Figure S2, respectively).

## 4 Discussion

In this proteomic profiling study, we investigated the association between 179 circulating protein biomarkers known to be associated with cardiovascular disease and the degree of PB and lipid core burden in 898 patients who underwent successful PCI after MI. LCBI and PB have recently been shown to be the strongest prospective identifiers of vulnerable non-flow limiting plaques associated with MACE [12]. A novel PEA technique was used to measure proteomic biomarkers chosen for their association with cardiovascular disease. Adjusted linear regression analysis showed a significant association of 24 proteins with PB, 28 proteins with LCBI and 8 with both PB and LCBI. After FDR correction, IL-18R1 and CSF-1 retained a statistically significant relationship with PB, and ANGPTL3 with LCBI.

Atherosclerosis is driven by lipid accumulation and a chronic inflammatory process within the arterial wall. Oxidized lipoproteins stimulate innate and adaptive immune responses leading to production of cytokines such as interleukins (IL) and colony-stimulating factors (CSF) playing a role in the pathologic process [17,18]. Previous studies have demonstrated positive associations between the cytokines IL1ra, IL-6, IL-12B, IL-18R1 and CSF-1 with either PB or LCBI [[19], [20], [21]].

We identified only one previous study examining associations between biomarkers and lipid core burden assessed by NIRS. In the study by Anroedh et al. [22], 26 different inflammatory and renal markers were analyzed in 203 patients; no significant associations were identified, possibly because the study was underpowered.

Several studies have investigated the relationship between circulating biomarkers and IVUS-derived plaque characteristics. In a study by Battes et al., higher TNF alpha (TNFα) levels were significantly associated with higher levels of IVUS-derived PB and presence of virtual histology-derived thin-cap fibroatheroma lesions (VH-TCFA) in 581 patients with stable angina pectoris [23]. Furthermore, lower levels of circulating IL-10 were associated with a higher risk of coronary PB and large VH-TCFA lesions. TNFα and IL-10 were included in our list of biomarkers, but no clear association was found between them and the plaque characteristics investigated.

After FDR correction, IL-18R1 and CSF-1 retained significant relationships with pan-coronary PB. IL-18 has been firmly established as a pro-atherosclerotic agent in the development of cardiovascular disease with studies suggesting a major role in atherosclerotic plaque destabilization [20,21]. It exerts its proinflammatory effects, such as interferon-gamma (IFNy)-inducing activities, after forming a signaling complex with IL-18R1, the ligand-binding chain in the IL-18 receptor [24,25]. In a study by Mallat et al., upregulation of IL-18R1 was noted in atherosclerotic plaques with and symptomatic patients compared to normal arteries [26]. Macrophage stimulating factor 1 (M-CSF1) is a cytokine known to play a central role in the inflammatory response in patients with acute coronary syndromes. Its primary function includes the regulation of growth and differentiation of monocytes and macrophages, both of which are elevated in patients with ACS [27,28]. Furthermore, studies in M-CSF1 deficit mice have shown that absence of M-CSF1 leads to a decrease in atherosclerosis development [29].

Lipid deposition is a hallmark of atherosclerosis. Therapeutic advances with statins and PCSK9 inhibitors have shown marked reduction of low-density lipoprotein cholesterol (LDL-C) and subsequent MACE [30,31]. Furthermore, plaque stabilization with reduced lipid core and slightly reduced PB has been shown after PCSK9-inhibition in the PACMAN and HUYGENS trials [32,33]. Accordingly, our results showed that PCSK9 had a significant positive association to both LCBI and PB.

Interestingly, ANGPTL3, an endogenous inhibitor of lipoprotein lipase (LPL) showed the strongest association with pan-coronary LCBI but not with PB, signaling an important role in lipid accumulation and vulnerable plaque development. Supporting this, we also observed a significant negative association between LPL levels and pan-coronary LCBI (p < 0.05). Deletion of the ANGPTL3 gene in ApoE-deficient mice has been reported to reduce the development of atherosclerosis [34]. Rare loss-of-function variants have also been shown to be associated with decreased LDL, cholesterol, and HDL levels [35,36]. RNA and antibody-based therapies inhibiting ANGPTL3 synthesis are in development and our findings indicate that they may be of value to reduce lipid deposition in the coronary arteries. Treatment with evinacumab, an antibody against ANGPTL3, has already been approved as treatment for pediatric patients with homozygous familial hypercholesterolemia, with successful results [37]. Furthermore, a novel genome editing tool is under development to permanently inactivate the ANGPTL3 gene in the liver to reduce LDL in patients with cardiovascular disease [38].

Apart from IL-18R1, CSF-1 and PCSK-9, 5 other proteins (VEGFA, EN-RAGE, CTSD, TR and OPN) had an unadjusted association to both LCBI and PB. Transferrin receptor levels increase during iron deficiency [39], a frequent comorbidity found in patients with ACS [40]. In the AtheroGene study, including 3423 patients with angiographically documented coronary heart disease, higher concentrations of TR were strongly associated with increased risk of myocardial infarction or cardiovascular death independently of CRP or hemoglobin [41]. Increased levels of VEGFA, a growth factor promoting angiogenesis [42] has been reported following acute MI, signaling the extent of myocardial damage [43,44] or alternatively expressing the potential for infarct healing.

EN-RAGE, also known as S100A12 is a proinflammatory copper binding protein [45] with enhanced expression during ACS, making it a potential marker of coronary plaque instability [46,47]. Notably, the same applies for OPN and CTSD, where studies measured increased expression in atheroma and post AMI [[48], [49], [50]].

We also examined the relationship between the biomarkers and MACE although this study was underpowered for hard outcomes. Several of the proteins associated with different aspects of morphology, such as CSF-1, IL-12B, EN-RAGE, PD-L1 and CTSZ were noted to have a significant association with adverse outcomes. In this study CSF-1 showed a strong relationship to both LCBI and PB. IL-12B, PD-L1, CTSZ with PB and PRTN3 and TIMP1 with LCBI.

A major strength of this study is the relatively large cohort, with data derived from extensive 3-vessel NIRS-IVUS imaging of coronary morphology in 3629 untreated non-culprit lesions from 898 patients. Imaging outcomes were measured in a blinded core lab, reducing bias and heterogeneity. Furthermore, the staff handling the biomarker data and the NIRS-IVUS outcomes were both blinded to each other's data, limiting data bias. In addition, the innovative proteomics method used in this study is highly sensitive and specific for the biomarkers chosen [14].

Limitations of this study include a population sample from Scandinavian countries only, limiting generalization to other lifestyles and countries NIRS and IVUS are not complication free modalities, yet we believe it carries minimal procedural risk when performed during clinically indicated coronary angiography as major imaging-related complications occurred in fewer than 1 % of our patients. Given its strong safety profile and mechanistic insights, we consider its use justifiable within the context of our study.

Another limitation is the lack of serial imaging outcomes and biomarker measurements over time. Additionally, we lack a validation cohort, as, to our knowledge, no other studies have combined PB and LCBI with biobanking in this manner.

## 5 Conclusions

In this study, we used an advanced proteomics technology to investigate the association between proteomics, cardiovascular disease and coronary morphology based on NIRS-IVUS-derived lipid core and PB in all three coronaries of patients with a recent MI. We were able to identify different biomarker patterns associated with PB and lipid rich vulnerable plaques. IL-18R1, and CSF-1 were shown to have a strong relationship with plaque burden. Only ANGPTL3 had an association with lipid rich plaques, but not with plaque burden, which further supports its role in lipid accumulation, development of vulnerable plaques and as a promising target for future therapeutics.

## CRediT authorship contribution statement

**Tania Sharma:** Writing – review & editing, Writing – original draft, Methodology, Investigation, Formal analysis, Data curation. **Akiko Maehara:** Writing – review & editing, Visualization, Data curation. **Michael Maeng:** Writing – review & editing, Project administration, Investigation. **Lars Kjøller-Hansen:** Writing – review & editing, Project administration, Investigation. **Thomas Engstrøm:** Writing – review & editing, Project administration, Investigation. **Ori Ben-Yehuda:** Writing – review & editing, Project administration, Investigation. **Mitsuaki Matsumura:** Writing – review & editing, Project administration, Investigation. **Ole Fröbert:** Writing – review & editing, Project administration, Investigation. **Jonas Persson:** Writing – review & editing, Project administration, Investigation. **Rune Wiseth:** Writing – review & editing, Project administration, Investigation. **Alf Inge Larsen:** Writing – review & editing, Project administration, Investigation. **Sasha Koul:** Writing – review & editing, Project administration, Investigation. **Rebecca Rylance:** Writing – review & editing, Methodology, Formal analysis. **Gary S. Mintz:** Writing – review & editing, Project administration, Investigation. **Ziad A. Ali:** Writing – review & editing, Project administration, Investigation. **Stefan K. James:** Writing – review & editing, Project administration, Investigation. **Gregg W. Stone:** Writing – review & editing, Visualization, Project administration, Methodology, Investigation, Formal analysis, Data curation, Conceptualization. **David Erlinge:** Writing – review & editing, Visualization, Validation, Supervision, Resources, Project administration, Methodology, Investigation, Funding acquisition, Formal analysis, Data curation, Conceptualization.

## Financial support

The analysis of the biomarkers was funded by Swedish Scientific Research Council and the Swedish Heart Lung Foundation.

## Declaration of competing interest

A.M. has received grant support and consultant fees from 10.13039/100011949Abbott Vascular and 10.13039/100008497Boston Scientific, and consultant fees for Conavi Medical Inc. Z.Z. reports no disclosures. O.BY. has received grants to the 10.13039/100007478Cardiovascular Research Foundation from Uppsala Clinical Research Center, 10.13039/501100005423Uppsala University Hospital, Uppsala, Sweden for core laboratory and data center analyses. M. Maeng reports support by a Borregaard Clinical Ascending Investigator grant from the 10.13039/501100009708Novo Nordisk Foundation (grant number NNF22OC0074083), has received lecture and/or advisory board fees from Astra-Zeneca, 10.13039/100004326Bayer, Boehringer-Ingelheim, 10.13039/100002491Bristol-Myers Squibb, and 10.13039/501100004191Novo Nordisk, has received research grants from 10.13039/100004320Philips, 10.13039/100004326Bayer and 10.13039/501100004191Novo Nordisk, has received a travel grant from 10.13039/501100004191Novo Nordisk, has ongoing institutional research contracts with 10.13039/100005565Janssen, 10.13039/501100004191Novo Nordisk and 10.13039/100004320Philips, and has equity interests in Eli 10.13039/100004312Lilly, 10.13039/501100004191Novo Nordisk, and Verve Therapeutics. J.P. reports institutional research grants from 10.13039/100011949Abbott Vascular. G.S.M. has received honoraria from 10.13039/100008497Boston Scientific, Medtronic, 10.13039/100004320Philips/Volcano, and 10.13039/501100008645Terumo. S.K.J reports personal fees from Medtronic and institutional research grants from Astra Zeneca, Jansen, 10.13039/100004326Bayer, 10.13039/100002429Amgen. Z.A.A. reports institutional research grants to St Francis Hospital from Abbott, 10.13039/100020297Abiomed, Acist Medical, 10.13039/100008497Boston Scientific, 10.13039/100016476Cardiovascular Systems Inc, Medtronic, 10.13039/100004320Philips, Opsens Medical, Telflex Inc; consultant honoraria from 10.13039/100002429Amgen, 10.13039/100004325AstraZeneca, 10.13039/100008497Boston Scientific., and equity in Shockwave. G.W.S has received speaker honoraria from Medtronic, Pulnovo, 10.13039/100020297Abiomed, 10.13039/100002429Amgen, Boehringer Ingelheim; has served as a consultant to Abbott, 10.13039/501100022274Daiichi Sankyo, Ablative Solutions, CorFlow, Cardiomech, Robocath, Miracor, Vectorious, 10.13039/100028184Apollo Therapeutics, Elucid Bio, Cardiac Success, Valfix, 10.13039/100019490TherOx, 10.13039/100020588HeartFlow, Neovasc, Ancora, Occlutech, 10.13039/100019443Impulse Dynamics, Adona Medical, Millennia Biopharma, Oxitope, HighLife, Elixir, Remote Cardiac Enablement, Aria; has equity/options from Cardiac Success, Ancora, Cagent, Applied Therapeutics, Biostar family of funds, SpectraWave, Orchestra Biomed, Aria, Valfix, Xenter; and his employer, Mount Sinai Hospital, receives research grants from Shockwave, Abbott, 10.13039/100020297Abiomed, Bioventrix, 10.13039/100016476Cardiovascular Systems Inc, 10.13039/100022632Phillips, Biosense-Webster, Vascular Dynamics, Pulnovo, V-wave and 10.13039/100006093PCORI (via 10.13039/100020424Weill Cornell Medical Center). D.E. reports personal fees from Astra Zeneca, 10.13039/100004326Bayer and 10.13039/100004339Sanofi. 10.13039/100024656TE reports speakers fee from 10.13039/100008497Boston Scientific, Abbott and 10.13039/501100004191Novo Nordisk as well as advisory board fee from Abbot and 10.13039/501100004191Novo Nordisk. The other authors declare no conflict of interests to the best of their knowledge.
