# Supplementary material for: Circulating protein biomarkers and their association with vulnerable plaque characteristics – a PROSPECT II substudy
Source: Int J Cardiol Cardiovasc Risk Prev. 2025 May 24;26:200440. doi: 10.1016/j.ijcrp.2025.200440 (PMC12155917; doi:10.1016/j.ijcrp.2025.200440)
Supplement: Multimedia component 1 [file mmc1.docx]

**Supplementary Appendix**

**Circulating protein biomarkers and their role in identifying vulnerable plaques**

Tania Sharma^1^, Akiko Maehara^2,3^, Ori Ben-Yehuda^3,4^, Michael Maeng^5^, Lars Kjoller-Hansen^6^, Thomas Engstrom^7^, Mitsuaki Matsumura^3^, Ole Frobert^8^, Jonas Persson^9^, Rune Wiseth^10^, Alf Inge Larsen^11^, Sasha Koul^1^, Rebecca Rylance^1^, Gary S. Mintz^3^, Ziad A. Ali^3^, Stefan K. James^12^, Gregg W. Stone^13^, David Erlinge^1,4^

1. Department of Cardiology, Clinical Sciences, Lund University, Lund, Sweden
2. New York-Presbyterian Hospital and Columbia University Irving Medical Center, New York, NY, USA
3. Cardiovascular Research Foundation, New York, NY, USA
4. University of California San Diego, San Diego, CA, USA
5. Aarhus University Hospital, Aarhus, Denmark
6. Zealand University Hospital, Roskilde, Denmark
7. University of Copenhagen, Copenhagen, Denmark
8. Orebro University, Faculty of Health, Orebro, Sweden
9. Danderyd University Hospital, Stockholm, Sweden
10. St Olavs Hospital, Trondheim, Norway
11. Stavanger University Hospital, Stavanger, Norway
12. Department of Medical Sciences and Uppsala Clinical Research Center, Uppsala University, Uppsala, Sweden
13. The Zena and Michael A. Wiener Cardiovascular Institute, Icahn School of Medicine at Mount Sinai, New York City, NY, USA

**Figure legends**

**Figure S1. Kaplan-Meier survival curves for non-culprit lesion-related major adverse cardiac events according to association with biomarkers divided by median**;

(A)CCL-16 (B)CD163 (C)CSF-1 (D)CTSZ (E)IL-10RB (F)IL-12B (G)IL-17RA (H)ITGB2 (I)KIT (J)LTBR (K)NOTCH3 (L)PD-L1 (M)PRTN3 (N)TIMP1 and (O)VASN.

Kaplan-Meier curves displaying the estimated survival probability for patients with recent MI in association with biomarker concentrations divided by median. These biomarkers were significant in the MACE analysis adjusted for age and sex. MACE includes the composite rate of cardiac death, myocardial infarction or unstable or progressive angina either requiring hospitalization or revascularization with rapid lesion progression.

**Table S1.**

**Patient clinical inclusion and exclusion criteria**

| **Clinical inclusion criteria (all must be present)** |
| --- |
| 1. Troponin positive ACS (STEMI >12 hours or NSTEMI) occurring within the prior 4 weeks of enrollment, with symptoms consistent with acute ischemia lasting >10 minutes, intended for angiography and PCI, if appropriate. |
| **Clinical exclusion criteria (none must be present)** |
| 1. Known estimated creatinine clearance <30 mL/min. 2. Cardiogenic shock, decompensated hypotension or heart failure requiring intubation, inotropes, intravenous diuretics, or a hemodynamic support device. 3. Patient has a known hypersensitivity, allergy, or contraindication to any of the following: Aspirin, both heparin and bivalirudin, all 3 of clopidogrel, prasugrel and ticagrelor, or to contrast that cannot be adequately pre-medicated. 4. Refractory ventricular arrhythmias (e.g. ventricular tachycardia or fibrillation) requiring either intravenous pharmacologic treatment or defibrillation during the index PCI procedure. 5. Persistent acute conduction system disease requiring temporary pacemaker insertion during the index PCI procedure. 6. Prior CABG at any time or planned CABG. 7. Any medical illness (e.g. cancer or severe congestive heart failure) or recent history of substance abuse that may cause non-compliance with the protocol (including follow-up angiography if enrolled in PROSPECT ABSORB), confound the data interpretation, or is associated with a life expectancy less than 3 years. 8. Patient is currently enrolled in another investigational use device or drug study that has not reached its primary endpoint. If the patient is enrolled in another study that is not investigational, required visits for that trial must not interfere with the conduct of this study. 9. Prior participation in this study. |

**Table S2.**

**Comparison of Baseline Characteristics Between High and Low Pan-Coronary Plaque Burden (PB) Groups**

| **Parameter** | **High PB, n = 448** | **Low PB, n = 429** | **P value** |
| --- | --- | --- | --- |
| Age (years) | 61.0 (32.0, 83.0) | 64.0 (32.0, 85.0) | 0.000 |
| Sex, female | 15.4% (69/448) | 19.1% (82/429) | 0.145 |
| Current or recent smoker | 27.38% (121/442) | 36.64(155/423) | 0.003 |
| Ex-smoker | 35.5% (157/442) | 27.42(116/423 | 0.010 |
| Never smoked | 37.1% (164/442) | 35.93(152/423 | 0.721 |
| Diabetes mellitus, all | 13.8% (62/448) | 10.3% (44/429) | 0.104 |
| Insulin-treated | 4.5% (20/448) | 4.2% (18/429) | 0.845 |
| Prior percutaneous coronary intervention | 14.1% (63/448) | 10.0% (43/429) | 0.067 |
| Hypertension requiring medication | 42.6% (191/448) | 31.7% (136/429) | 0.001 |
| Hyperlipidemia requiring medication | 27.7% (124/448) | 22.6% (97/429) | 0.084 |
| Clinical presentation |  |  |  |
| ST-segment elevation myocardial infarction | 24.3% (109/448) | 20.0% (86/429) | 0.127 |
| Non-ST-segment elevation myocardial infarction | 75.7% (339/448) | 80.0% (343/429) | 0.127 |

Continuous variables are presented as median (Q1, Q3). and compared using the Mann–Whitney U test. Categorical variables are presented as percentage and compared using the chi-squared test. A two-sided p-value of <0.05 was considered statistically significant.

**Table S3.**

**Comparison of Baseline Characteristics Between High and Low Pan-Coronary Lipid Core Burden Index (LCBI) Groups**

| **Parameter** | **High LCBI, n = 446** | **Low LCBI, n = 431** | **P value** |
| --- | --- | --- | --- |
| Age (years) | 63.0 (32.0, 85.0) | 63.0 (32.0, 85.0) | 0.03 |
| Sex, female | 19.1% (85/446) | 15.3% (66/431) | 0.142 |
| Current or recent smoker | 30.1% (132/438) | 33.7% (144/427) | 0.258 |
| Ex-smoker | 35.6% (156/438) | 27.4% (117/427) | 0.009 |
| Never smoked | 34.3% (150/438) | 38.9% (166/427) | 0.157 |
| Diabetes mellitus, all | 12.3% (55/446) | 11.8% (51/431) | 0.821 |
| Insulin-treated | 4.7% (21/446) | 3.9% (17/431) | 0.578 |
| Prior percutaneous coronary intervention | 12.1% (54/446) | 12.1% (52/431) | 0.985 |
| Hypertension requiring medication | 39.7% (177/446) | 34.8% (150/431) | 0.135 |
| Hyperlipidemia requiring medication | 24.9% (111/446) | 25.5% (110/431) | 0.829 |
| Clinical presentation |  |  |  |
| ST-segment elevation myocardial infarction | 24.2% (108/446) | 20.2% (87/431) | 0.151 |
| Non-ST-segment elevation myocardial infarction | 75.8% (338/446) | 79.8% (344/431) | 0.151 |

Continuous variables are presented as median (Q1, Q3). and compared using the Mann–Whitney U test. Categorical variables are presented as percentage and compared using the chi-squared test. A two-sided p-value of <0.05 was considered statistically significant.

**Table S4.**

| **179 circulating protein biomarkers measured** | |
| --- | --- |
| ACE2 | Angiotensin-converting enzyme 2 |
| ADA | Adenosine Deaminase |
| ALCAM | CD166 antigen |
| AMBP | Protein AMBP |
| ANG | Angiogenin |
| ANGPT1 | Angiopoietin-1 |
| ANGPTL3 | Angiopoietin-related protein 3 |
| AOC3 | Membrane primary amine oxidase |
| AP-N | Aminopeptidase N |
| AXL | Tyrosine-protein kinase receptor UFO |
| AZU1 | Azurocidin |
| BNP | Natriuretic peptides B |
| CASP-3 | Caspase-3 |
| CCL11 | Eotaxin |
| CCL15 | C-C motif chemokine 15 |
| CCL16 | C-C motif chemokine 16 |
| CCL19 | C-C motif chemokine 19 |
| CCL20 | C-C motif chemokine 20 |
| CCL23 | C-C motif chemokine 23 |
| CCL24 | C-C motif chemokine 24 |
| CCL25 | C-C motif chemokine 25 |
| CCL3 | C-C motif chemokine 3 |
| CCL4 | C-C motif chemokine 4 |
| CD163 | Scavenger receptor cysteine-rich type 1 protein |
| CD40 | CD40L receptor |
| CD59 | CD59 glycoprotein |
| CD6 | T cell surface glycoprotein CD6 isoform |
| CD8A | T-cell surface glycoprotein CD8 alpha chain |
| CD93 | Complement component C1q receptor |
| CDH1 | Cadherin-1 |
| CDH5 | Cadherin-5 |
| CHI3L1 | Chitinase-3-like protein 1 |
| CHIT1 | Chitotriosidase-1 |
| CHL1 | Neural cell adhesion molecule L1-like protein |
| CNTN1 | Contactin-1 |
| COL18A1 | Collagen alpha-1(XVIII) chain |
| COL1A1 | Collagen alpha-1(I) chain |
| COMP | Cartilage oligomeric matrix protein |
| CSF-1 | Macrophage colony-stimulating factor 1 |
| CTSD | Cathepsin D |
| CTSL1 | Cathepsin L1 |
| CTSZ | Cathepsin Z |
| CXCL1 | C-X-C motif chemokine 1 |
| CXCL10 | C-X-C motif chemokine 10 |
| CXCL11 | C-X-C motif chemokine 11 |
| CXCL5 | C-X-C motif chemokine 5 |
| CXCL6 | C-X-C motif chemokine 6 |
| DCN | Decorin |
| DECR1 | 2,4-dienoyl-CoA reductase, mitochondrial |
| DPP4 | Dipeptidyl peptidase 4 |
| EN-RAGE | Protein S100-A12 |
| ENG | Endoglin |
| Ep-CAM | Epithelial cell adhesion molecule |
| EPHB4 | Ephrin type-B receptor 4 |
| FABP2 | Fatty acid-binding protein, intestinal |
| FABP4 | Fatty acid-binding protein, adipocyte |
| FAS | Tumor necrosis factor receptor superfamily member 6 |
| FGF-21 | Fibroblast growth factor 21 |
| Gal-3 | Galectin-3 |
| Gal-4 | Galectin-4 |
| Gal-9 | Galectin-9 |
| GAS6 | Growth arrest-specific protein 6 |
| GDF-2 | Growth/differentiation factor 2 |
| GT | Gastrotropin |
| HAOX1 | Hydroxyacid oxidase 1 |
| HO-1 | Heme oxygenase 1 |
| HSP 27 | Heat shock 27 kDa protein |
| ICAM2 | Intercellular adhesion molecule 2 |
| ICAM1 | Intercellular adhesion molecule 2 |
| ICAM3 | Intercellular adhesion molecule 2 |
| IDUA | Alpha-L-iduronidase |
| IGFBP-2 | Insulin-like growth factor-binding protein 2 |
| IGFBP-7 | Insulin-like growth factor-binding protein 7 |
| IGFBP3 | Insulin-like growth factor-binding protein 3 |
| IL-10RB | Interleukin-10 receptor subunit beta |
| IL-12B | Interleukin-12 subunit beta |
| IL-17A | Interleukin-17A |
| IL-17RA | Interleukin-17 receptor A |
| IL-18R1 | Interleukin-18 receptor 1 |
| IL-1ra | Interleukin-1 receptor antagonist protein |
| IL-1RT1 | Interleukin-1 receptor type 1 |
| IL-20RA | Interleukin-20 receptor subunit alpha |
| IL-6RA | Interleukin-6 receptor subunit alpha |
| IL-10 | Interleukin-10 |
| IL-18 | Interleukin-18 |
| IL-1RL2 | Interleukin-1 receptor-like 2 |
| IL-2RA | Interleukin-2 receptor subunit alpha |
| IL-6 | Interleukin-6 |
| IL-8 | Interleukin-8 |
| ITGAM | Integrin alpha-M |
| ITGB2 | Integrin beta-2 |
| JAM-A | Junctional adhesion molecule A |
| KIT | Mast/stem cell growth factor receptor Kit |
| LAP TGF-beta-1 | Latency-associated peptide transforming growth factor beta-1 |
| LCN2 | Neutrophil gelatinase-associated lipocalin |
| LDL receptor | Low-density lipoprotein receptor |
| LEP | Leptin |
| LOX-1 | Lectin-like oxidized LDL receptor 1 |
| LPL | Lipoprotein lipase |
| LTBR | Lymphotoxin-beta receptor |
| LYVE1 | Lymphatic vessel endothelial hyaluronic acid receptor 1 |
| MB | Myoglobin |
| MCP-1 | Monocyte chemotactic protein 1 |
| MCP-2 | Monocyte chemotactic protein 2 |
| MCP-3 | Monocyte chemotactic protein 3 |
| MCP-4 | Monocyte chemotactic protein 4 |
| MMP-2 | Matrix metalloproteinase-2 |
| MMP-3 | Matrix metalloproteinase-3 |
| MMP-9 | Matrix metalloproteinase-9 |
| MMP-12 | Matrix metalloproteinase-12 |
| MMP-7 | Matrix metalloproteinase-7 |
| MPO | Myeloperoxidase |
| NCAM1 | Neural cell adhesion molecule 1 |
| NID1 | Nidogen-1 |
| NOTCH3 | Neurogenic locus notch homolog protein 3 |
| NOTCH1 | Neurogenic locus notch homolog protein 1 |
| NRP1 | Neuropilin-1 |
| OPG | Osteoprotegerin |
| OPN | Osteopontin |
| OSM | Oncostatin-M |
| PAI | Plasminogen activator inhibitor 1 |
| PAM | Peptidyl-glycine alpha-amidating monooxygenase |
| PCSK9 | Proprotein convertase subtilisin/kexin type 9 |
| PD-L1 | Programmed cell death 1 ligand 1 |
| PDGF subunit A | Platelet-derived growth factor subunit A |
| PDGF subunit B | Platelet-derived growth factor subunit B |
| PECAM-1 | Platelet endothelial cell adhesion molecule |
| PGF | Placenta growth factor |
| PGLYRP1 | Peptidoglycan recognition protein 1 |
| PLC | Perlecan |
| PLXNB2 | Plexin-B2 |
| PON3 | Paraoxonase |
| PRELP | Prolargin |
| PROC | Vitamin K-dependent protein C |
| PRSS2 | Trypsin-2 |
| PRTN3 | Myeloblastin |
| PSGL-1 | P-selectin glycoprotein ligand 1 |
| PSP-D | Pulmonary surfactant-associated protein D |
| PTPRS | Receptor-type tyrosine-protein phosphatase S |
| RAGE | Receptor for advanced glycosylation end products |
| RARRES2 | Retinoic acid receptor responder protein 2 |
| REG3A | Regenerating islet-derived protein 3-alpha |
| SELE | E-selectin |
| SELL | L-selectin |
| SELP | P-selectin |
| SHPS-1 | Tyrosine-protein phosphatase non-receptor type substrate 1 |
| SIRT2 | SIR2-like protein 2 |
| SPON1 | Spondin-1 |
| ST2 | ST2 protein |
| ST6GAL1 | Beta-galactoside alpha-2,6-sialyltransferase 1 |
| STAMBP | STAM-binding protein |
| STK4 | Serine/threonine-protein kinase 4 |
| TGFBI | Transforming growth factor-beta-induced protein ig-h3 |
| TGFBR3 | Transforming growth factor beta receptor type 3 |
| TGM2 | Protein-glutamine gamma-glutamyltransferase 2 |
| THBS4 | Thrombospondin-4 |
| TIE1 | Tyrosine-protein kinase receptor Tie-1 |
| TIE2 | Angiopoietin-1 receptor |
| TIMP1 | Metalloproteinase inhibitor 1 |
| TIMP4 | Metalloproteinase inhibitor 4 |
| TLT-2 | Trem-like transcript 2 protein |
| TNC | Tenascin |
| TNFα | Tumor necrosis factor alpha |
| TNF-R1 | Tumor necrosis factor receptor 1 |
| TNF-R2 | Tumor necrosis factor receptor 2 |
| TNFB | TNF-beta |
| TNFRSF10C | Tumor necrosis factor receptor superfamily member 10C |
| TNFRSF14 | Tumor necrosis factor receptor superfamily member 14 |
| TNFRSF9 | Tumor necrosis factor receptor superfamily member 9 |
| TNXB | Tenascin-X |
| TR | Transferrin receptor protein 1 |
| TR-AP | Tartrate-resistant acid phosphatase type 5 |
| TRAIL | TNF-related apoptosis-inducing ligand |
| TRANCE | TNF-related activation-induced cytokine |
| TWEAK | Tumor necrosis factor (Ligand) superfamily, member 12 |
| UMOD | Uromodulin |
| VASN | Vasorin |
| VCAM1 | Vascular cell adhesion protein 1 |
| VEGFA | Vascular endothelial growth factor A |
| VEGFD | Vascular endothelial growth factor D |
|  |  |

**Table S5.**

| **Adjusted Linear Regression results of Biomarkers and Imaging Outcomes** | | | | |
| --- | --- | --- | --- | --- |
|  | **PB** | | **LCBI** | |
| **Biomarker** | **coefficient** | **p value** | **coefficient** | **p value** |
| ACE2 | 0.676 | 0.069 | 2.931 | 0.212 |
| ADA | 0.640 | 0.147 | 3.919 | 0.173 |
| ALCAM | 0.128 | 0.846 | -0.665 | 0.875 |
| AMBP | 0.755 | 0.587 | 6.963 | 0.427 |
| ANG | 0.173 | 0.672 | 5.074 | 0.053 |
| ANGPT1 | -0.133 | 0.494 | 0.312 | 0.799 |
| ANGPTL3 | 0.053 | 0.917 | 11.011 | 0.001* |
| AOC3 | 0.095 | 0.877 | -0.146 | 0.970 |
| AP-N | 0.071 | 0.916 | 0.453 | 0.916 |
| AXL | 0.573 | 0.275 | -2.288 | 0.496 |
| AZU1 | -0.138 | 0.454 | -2.767 | 0.019* |
| BNP | 0.045 | 0.767 | 2.709 | 0.006* |
| CASP-3 | 0.166 | 0.269 | 0.848 | 0.379 |
| CCL11 | -0.431 | 0.395 | -1.518 | 0.641 |
| CCL15 | 0.494 | 0.243 | 0.777 | 0.777 |
| CCL16 | 0.645 | 0.115 | 2.217 | 0.399 |
| CCL19 | 0.668 | 0.021* | 2.890 | 0.122 |
| CCL20 | -0.004 | 0.987 | -0.930 | 0.592 |
| CCL23 | 0.819 | 0.066 | 8.763 | 0.002* |
| CCL24 | 0.425 | 0.083 | -1.628 | 0.301 |
| CCL25 | -0.204 | 0.521 | -2.165 | 0.288 |
| CCL3 | 0.409 | 0.227 | 2.155 | 0.322 |
| CCL4 | 0.416 | 0.187 | 2.441 | 0.228 |
| CD163 | 0.782 | 0.061 | -0.383 | 0.886 |
| CD40 | 0.910 | 0.069 | 4.316 | 0.179 |
| CD59 | 0.425 | 0.424 | 6.541 | 0.055 |
| CD6 | 0.092 | 0.810 | -3.872 | 0.115 |
| CD8A | 0.537 | 0.093 | -1.820 | 0.374 |
| CD93 | -0.306 | 0.622 | -2.246 | 0.575 |
| CDH1 | 0.262 | 0.614 | 2.188 | 0.515 |
| CDH5 | -0.565 | 0.309 | -7.579 | 0.034* |
| CHI3L1 | 0.349 | 0.186 | 6.065 | 0.000* |
| CHIT1 | 0.090 | 0.727 | 3.845 | 0.020* |
| CHL1 | -0.082 | 0.897 | 9.427 | 0.021* |
| CNTN1 | -0.208 | 0.726 | -6.486 | 0.088 |
| COL18A1 | 0.637 | 0.240 | 6.307 | 0.070 |
| COL1A1 | -0.452 | 0.434 | -5.524 | 0.137 |
| COMP | -0.312 | 0.518 | 1.642 | 0.597 |
| CSF-1 | 3.336 | 0.000* | 11.273 | 0.049* |
| CTSD | 1.414 | 0.004* | 7.624 | 0.016* |
| CTSL1 | 1.183 | 0.073 | 6.435 | 0.121 |
| CTSZ | 1.271 | 0.038* | 5.633 | 0.153 |
| CXCL1 | 0.030 | 0.880 | 1.424 | 0.268 |
| CXCL10 | 0.227 | 0.316 | -0.195 | 0.894 |
| CXCL11 | -0.066 | 0.786 | 0.760 | 0.629 |
| CXCL5 | -0.016 | 0.904 | 0.585 | 0.481 |
| CXCL6 | 0.166 | 0.517 | 0.330 | 0.841 |
| DCN | 0.141 | 0.884 | -7.554 | 0.215 |
| DECR1 | 0.091 | 0.582 | 0.456 | 0.662 |
| DPP4 | -0.229 | 0.686 | -0.047 | 0.990 |
| EN-RAGE | 0.940 | 0.002* | 4.820 | 0.015* |
| ENG | -1.012 | 0.177 | -2.439 | 0.614 |
| Ep-CAM | -0.305 | 0.198 | -0.977 | 0.521 |
| EPHB4 | 0.169 | 0.788 | 2.421 | 0.549 |
| FABP2 | -0.077 | 0.735 | -1.034 | 0.472 |
| FABP4 | 0.086 | 0.771 | 1.667 | 0.379 |
| FAS | 0.906 | 0.066 | 1.951 | 0.537 |
| FGF-21 | -0.043 | 0.798 | 1.587 | 0.141 |
| Gal-3 | 0.312 | 0.555 | 3.450 | 0.312 |
| Gal-4 | 0.403 | 0.267 | -1.461 | 0.532 |
| Gal-9 | 1.544 | 0.042* | -1.816 | 0.705 |
| GAS6 | 0.575 | 0.329 | 5.417 | 0.152 |
| GDF-2 | -0.288 | 0.480 | 1.884 | 0.463 |
| GT | -0.394 | 0.232 | -0.820 | 0.692 |
| HAOX1 | -0.138 | 0.377 | -0.708 | 0.474 |
| HO-1 | 0.503 | 0.431 | 9.342 | 0.020* |
| HSP 27 | 0.474 | 0.333 | 1.237 | 0.690 |
| ICAM-2 | -0.280 | 0.587 | -2.183 | 0.509 |
| ICAM1 | 0.408 | 0.478 | 1.950 | 0.598 |
| ICAM3 | 0.498 | 0.387 | 2.966 | 0.421 |
| IDUA | 0.193 | 0.674 | 4.100 | 0.158 |
| IGFBP-2 | -0.701 | 0.024* | 3.387 | 0.091 |
| IGFBP-7 | 0.728 | 0.123 | 1.897 | 0.532 |
| IGFBP3 | -0.358 | 0.462 | 0.598 | 0.848 |
| IL-10RB | 1.062 | 0.121 | 3.190 | 0.470 |
| IL-12B | 0.849 | 0.010* | -1.140 | 0.591 |
| IL-17A | 0.089 | 0.779 | -0.451 | 0.826 |
| IL-17RA | 0.120 | 0.760 | -1.539 | 0.540 |
| IL-18R1 | 2.157 | 0.000* | 8.031 | 0.022* |
| IL-1ra | 1.004 | 0.003* | 2.981 | 0.164 |
| IL-1RT1 | -0.077 | 0.898 | -0.375 | 0.922 |
| IL-20RA | -0.151 | 0.807 | -1.580 | 0.684 |
| IL-6RA | 0.226 | 0.672 | -3.581 | 0.296 |
| IL10 | 0.241 | 0.428 | -0.197 | 0.920 |
| IL18 | 0.379 | 0.362 | -1.823 | 0.495 |
| IL1RL2 | -0.746 | 0.200 | -6.792 | 0.064 |
| IL2-RA | 0.367 | 0.441 | 0.800 | 0.793 |
| IL6 | 0.223 | 0.237 | 3.050 | 0.011* |
| IL8 | -0.171 | 0.627 | 2.441 | 0.280 |
| ITGAM | 0.416 | 0.454 | -1.641 | 0.612 |
| ITGB2 | 0.656 | 0.114 | -3.261 | 0.221 |
| JAM-A | 0.314 | 0.131 | 1.133 | 0.397 |
| KIT | -0.868 | 0.153 | -5.727 | 0.142 |
| LAP TGF-beta-1 | 1.006 | 0.044* | 4.461 | 0.165 |
| LCN2 | 0.494 | 0.222 | 0.294 | 0.911 |
| LDL receptor | -0.055 | 0.887 | 1.903 | 0.442 |
| LEP | 0.187 | 0.483 | 0.828 | 0.625 |
| LOX-1 | 0.330 | 0.338 | 0.027 | 0.990 |
| LPL | -0.748 | 0.133 | -7.348 | 0.019* |
| LTBR | 0.940 | 0.097 | 4.958 | 0.176 |
| LYVE1 | -0.059 | 0.915 | 0.297 | 0.933 |
| MB | -0,605 | 0.023* | -2,705 | 0.114 |
| MCP-1 | -0.295 | 0.386 | -1.021 | 0.640 |
| MCP-2 | 0.282 | 0.408 | 2.555 | 0.242 |
| MCP-3 | 0.837 | 0.060 | 8.448 | 0.003* |
| MCP-4 | -0.349 | 0.177 | -0.996 | 0.547 |
| MMP-2 | 0.425 | 0.453 | 3.819 | 0.293 |
| MMP-3 | 0.422 | 0.243 | 4.295 | 0.065 |
| MMP-9 | 0.640 | 0.030* | 2.550 | 0.183 |
| MMP12 | 0.327 | 0.320 | 4.135 | 0.046* |
| MMP7 | 0.134 | 0.715 | 1.836 | 0.433 |
| MPO | -0.022 | 0.954 | -6.204 | 0.011* |
| NCAM1 | -0.049 | 0.935 | -0.002 | 1.000 |
| NID1 | 0.051 | 0.913 | 5.375 | 0.074 |
| Notch 3 | -0.400 | 0.466 | -1.656 | 0.640 |
| NOTCH1 | 0.485 | 0.542 | -0.197 | 0.969 |
| NRP1 | 0.321 | 0.712 | 3.262 | 0.559 |
| OPG | -0.334 | 0.411 | -1.053 | 0.688 |
| OPN | 0.746 | 0.045* | 5.704 | 0.017* |
| OSM | 0.611 | 0.014* | 3.108 | 0.055 |
| PAI | 0.206 | 0.376 | 1.827 | 0.222 |
| PAM | 0.326 | 0.641 | 5.037 | 0.262 |
| PCSK9 | 1.635 | 0.008* | 8.559 | 0.031* |
| PD-L1 | 1.511 | 0.007* | 3.528 | 0.327 |
| PDGF subunit A | -0.046 | 0.845 | 1.013 | 0.499 |
| PDGF subunit B | -0.096 | 0.572 | 0.504 | 0.639 |
| PECAM-1 | 0.268 | 0.386 | -0.115 | 0.954 |
| PGF | 1.472 | 0.026* | 4.535 | 0.280 |
| PGLYRP1 | 0.706 | 0.067 | 2.722 | 0.276 |
| PLC | 0.382 | 0.517 | -0.167 | 0.965 |
| PLXNB2 | 0.789 | 0.328 | -0.808 | 0.876 |
| PON3 | -0.959 | 0.004* | -2.233 | 0.305 |
| PRELP | -1.243 | 0.222 | -0.882 | 0.891 |
| PROC | 0.437 | 0.397 | 5.872 | 0.076 |
| PRSS2 | 0.602 | 0.098 | 6.238 | 0.007* |
| PRTN3 | 0.761 | 0.010* | 1.340 | 0.484 |
| PSGL-1 | 0.439 | 0.608 | -2.885 | 0.592 |
| PSP-D | -0.230 | 0.439 | -0.451 | 0.813 |
| PTPRS | -1.178 | 0.119 | 3.536 | 0.466 |
| RAGE | -0.957 | 0.047* | -1.337 | 0.661 |
| RARRES2 | 0.910 | 0.186 | 3.350 | 0.449 |
| REG3A | -1.595 | 0.665 | 0.042 | 0.999 |
| SELE | 0.099 | 0.788 | -2.820 | 0.234 |
| SELL | -0.043 | 0.941 | -1.243 | 0.738 |
| SELP | 0.099 | 0.726 | 0.070 | 0.969 |
| SHPS-1 | 0.826 | 0.098 | 2.420 | 0.451 |
| SIRT2 | 0.249 | 0.156 | 0.292 | 0.798 |
| SPON1 | -0.424 | 0.037* | -1.888 | 0.143 |
| ST2 | 0.258 | 0.447 | 5.408 | 0.014* |
| ST6GAL1 | 0.860 | 0.073 | 7.962 | 0.010* |
| STAMBP | 0.381 | 0.074 | 0.573 | 0.677 |
| STK4 | 0.240 | 0.220 | 0.555 | 0.660 |
| TGFBI | 0.227 | 0.670 | 5.011 | 0.144 |
| TGFBR3 | -0.558 | 0.329 | 0.719 | 0.844 |
| TGM2 | -0.059 | 0.867 | -3.652 | 0.105 |
| THBS4 | 0.215 | 0.598 | 1.061 | 0.687 |
| TIE1 | 0.217 | 0.784 | 3.136 | 0.540 |
| TIE2 | -0.073 | 0.928 | -7.801 | 0.132 |
| TIMP1 | 0.584 | 0.244 | 10.201 | 0.002* |
| TIMP4 | -0.725 | 0.126 | 5.743 | 0.058 |
| TLT-2 | 0.254 | 0.541 | -3.785 | 0.155 |
| TNC | -0.043 | 0.909 | 6.655 | 0.006* |
| TNFα | 0.787 | 0.084 | 0.955 | 0.748 |
| TNF-R1 | 0.835 | 0.082 | 3.784 | 0.222 |
| TNF-R2 | 0.870 | 0.059 | 1.871 | 0.528 |
| TNFB | 0.500 | 0.248 | -2.361 | 0.396 |
| TNFRSF10C | 0.354 | 0.374 | -1.053 | 0.681 |
| TNFRSF14 | 0.722 | 0.109 | 1.188 | 0.681 |
| TNFRSF9 | 0.875 | 0.072 | 0.511 | 0.871 |
| TNXB | -0.580 | 0.482 | 3.432 | 0.516 |
| TR | 0.923 | 0.017* | 8.567 | 0.001* |
| TR-AP | 0.312 | 0.587 | 1.282 | 0.729 |
| TRAIL | 0.474 | 0.453 | 0.615 | 0.879 |
| TRANCE | 0.239 | 0.547 | -2.563 | 0.315 |
| TWEAK | -0.259 | 0.092 | -2.161 | 0.028* |
| UMOD | 0.275 | 0.783 | 1.729 | 0.790 |
| VASN | -0.078 | 0.917 | 2.442 | 0.610 |
| VCAM1 | 1.043 | 0.083 | 1.108 | 0.776 |
| VEGFA | 1.987 | 0.002* | 12.148 | 0.003* |
| VEGFD | -0.052 | 0.919 | -5.228 | 0.106 |
|  |  |  |  |  |

*Significant after false discovery rate correction (p value < 0.05)

**Figure S1 A**

**
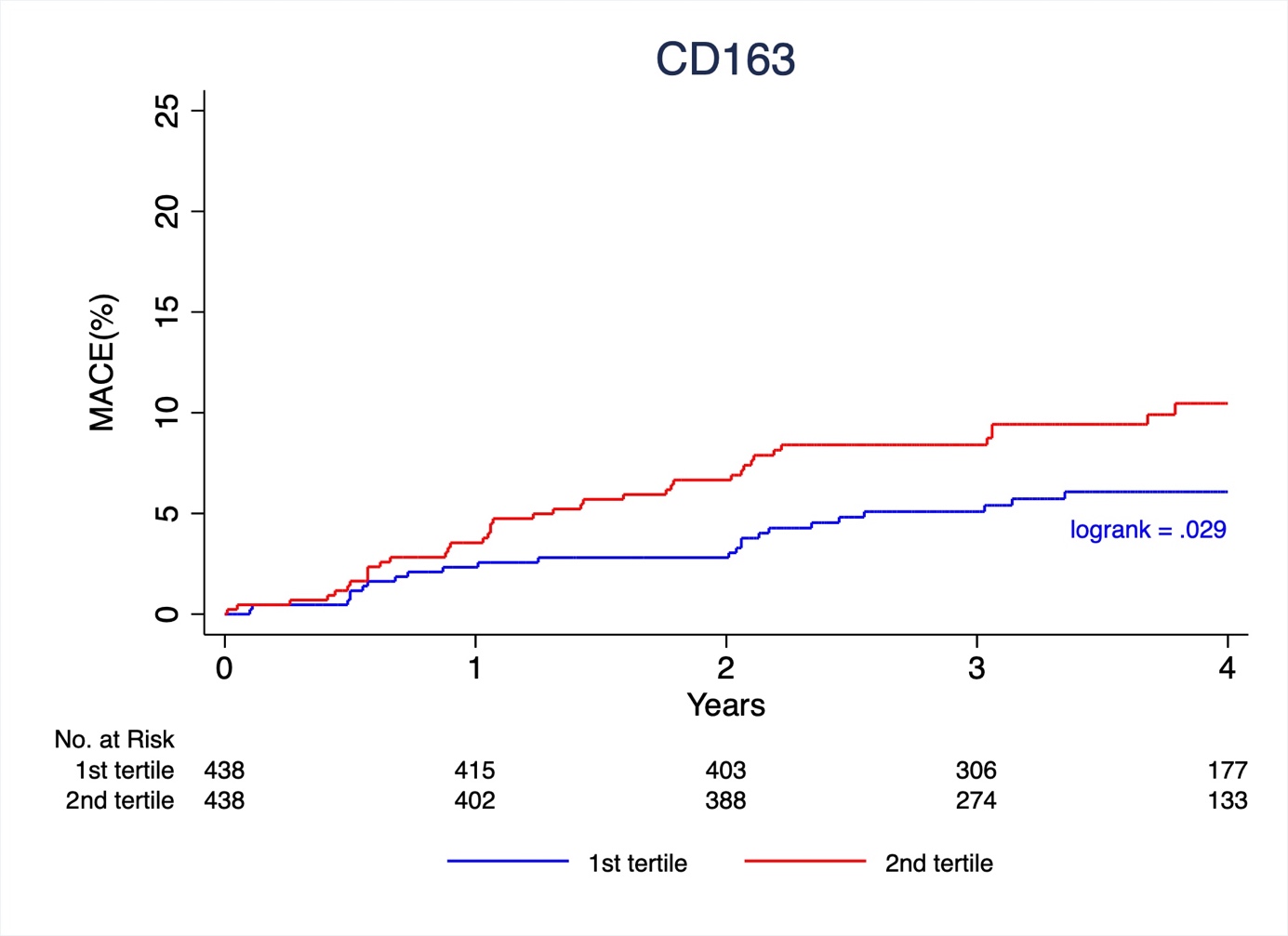

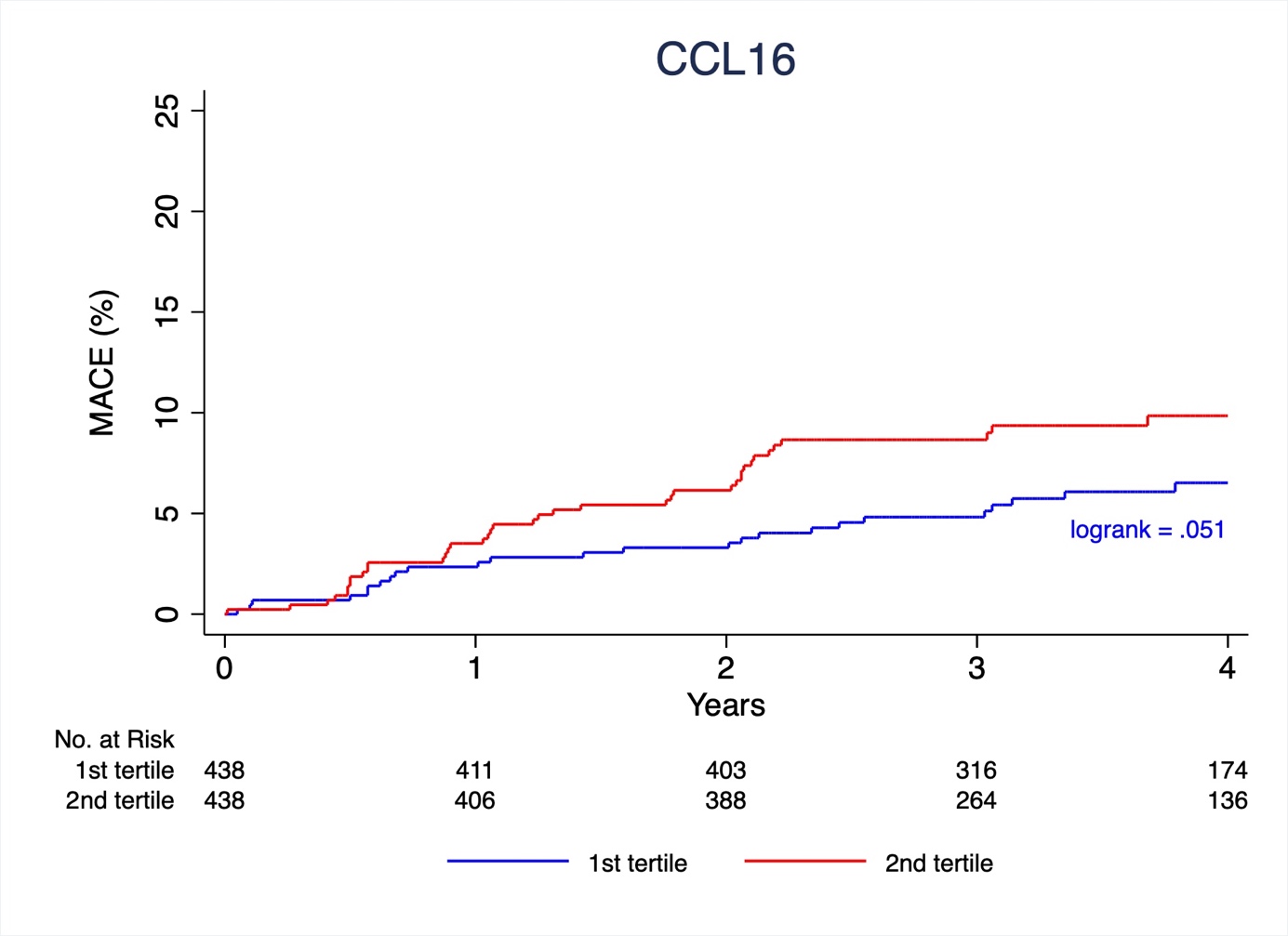
Figure S1 B**

**Figure S1 C**

**
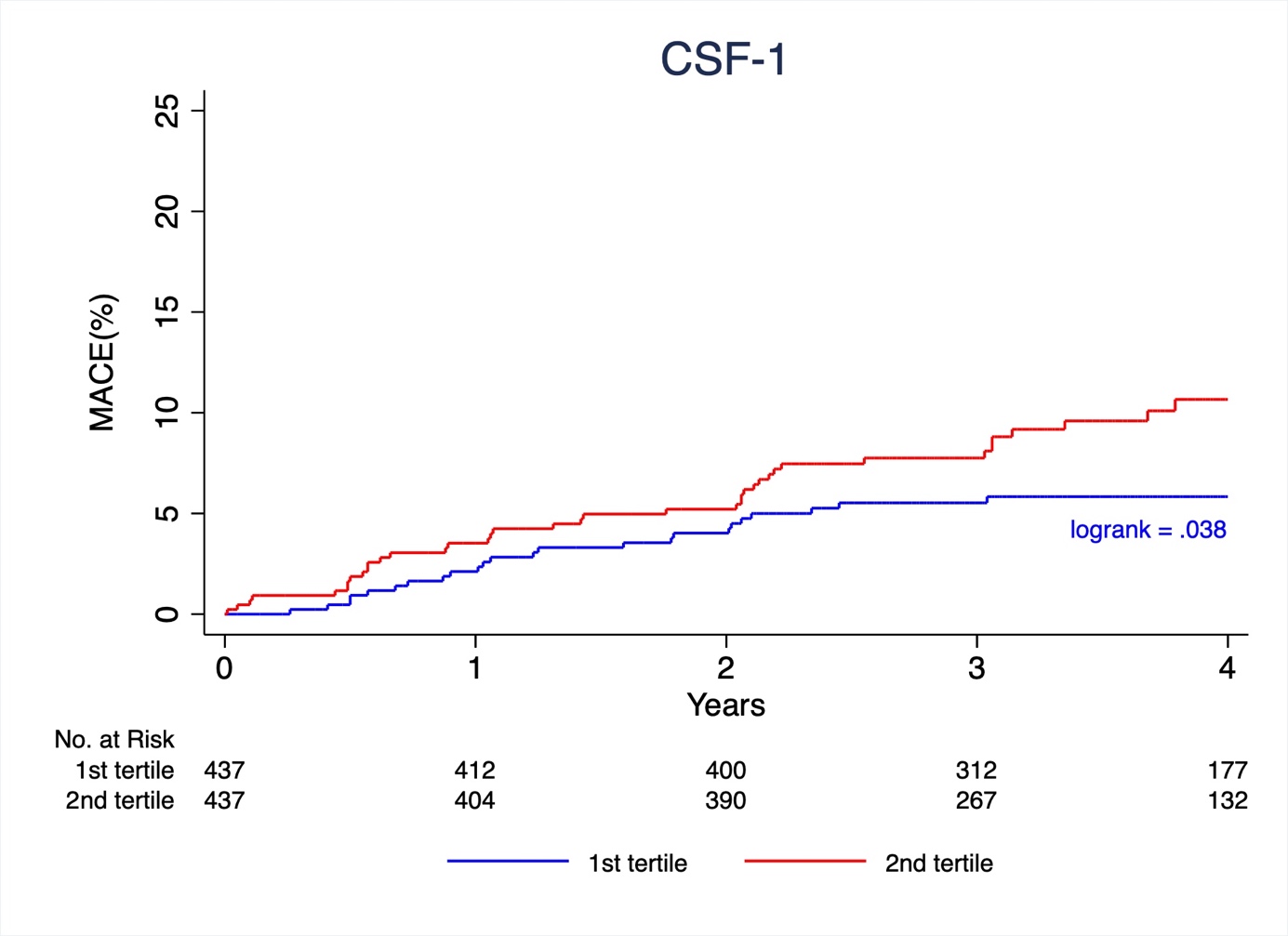
**

**
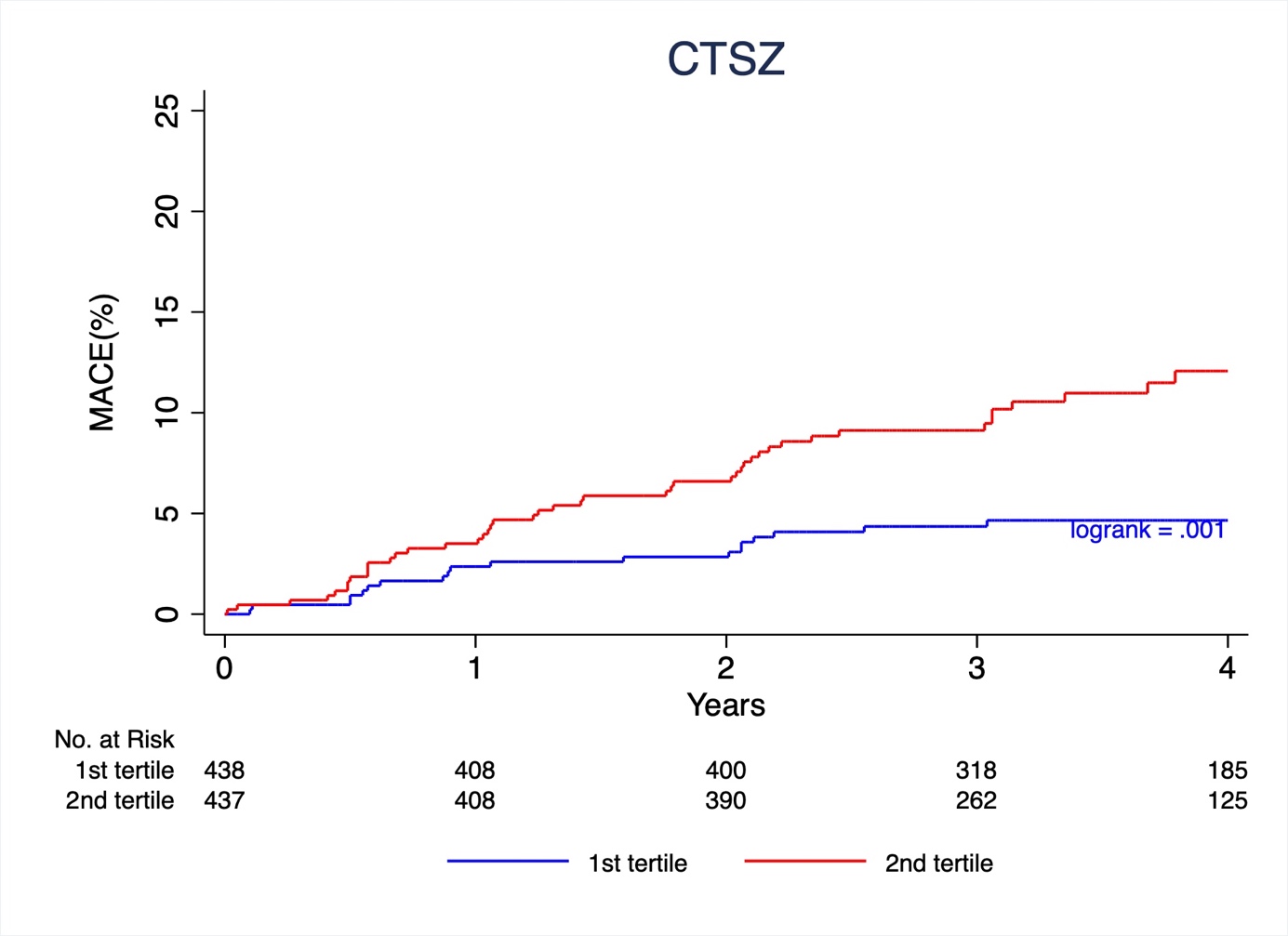
Figure S1 D**

**Figure S1 E**

**
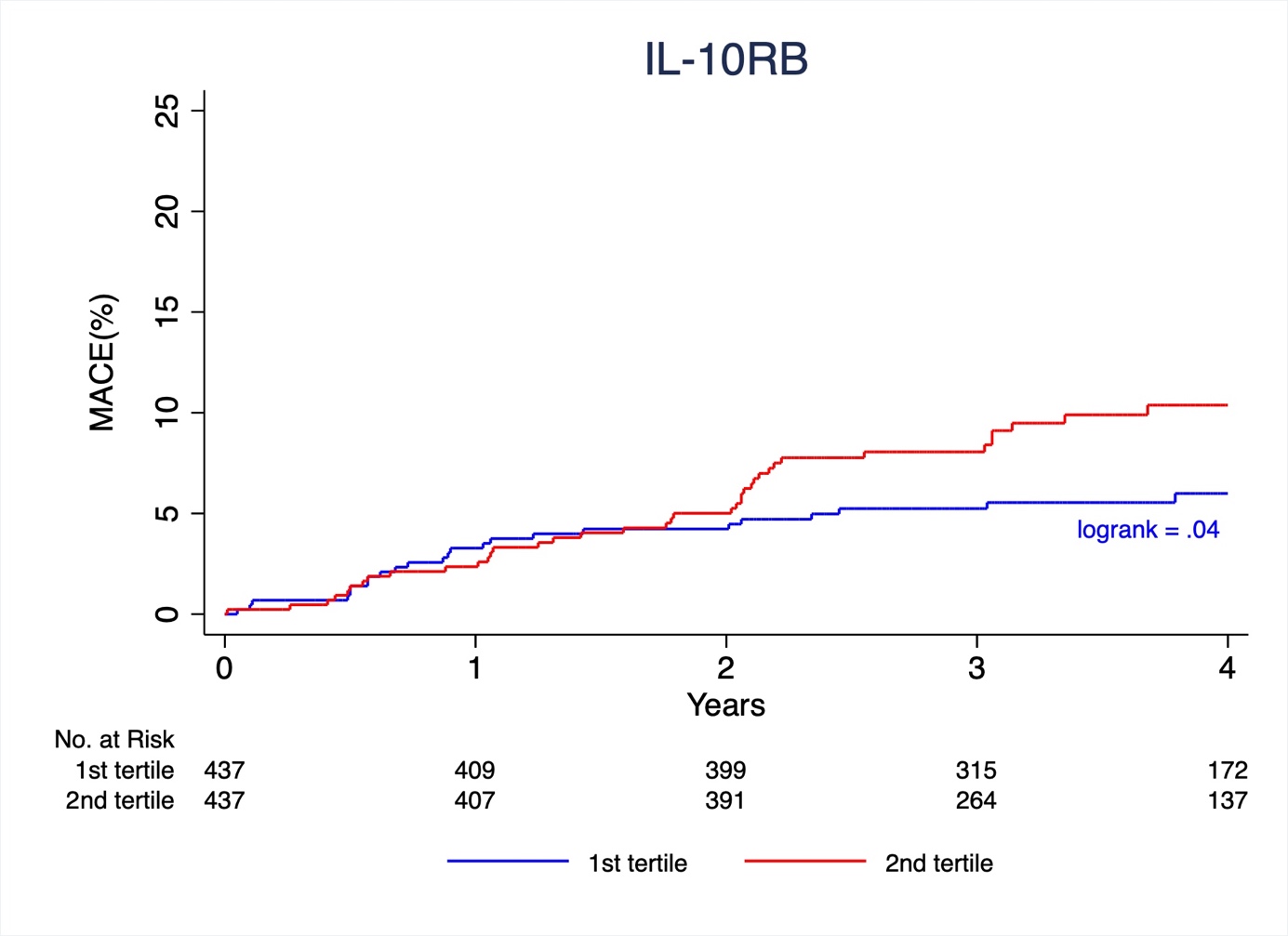
**

**Figure S1 F**

**
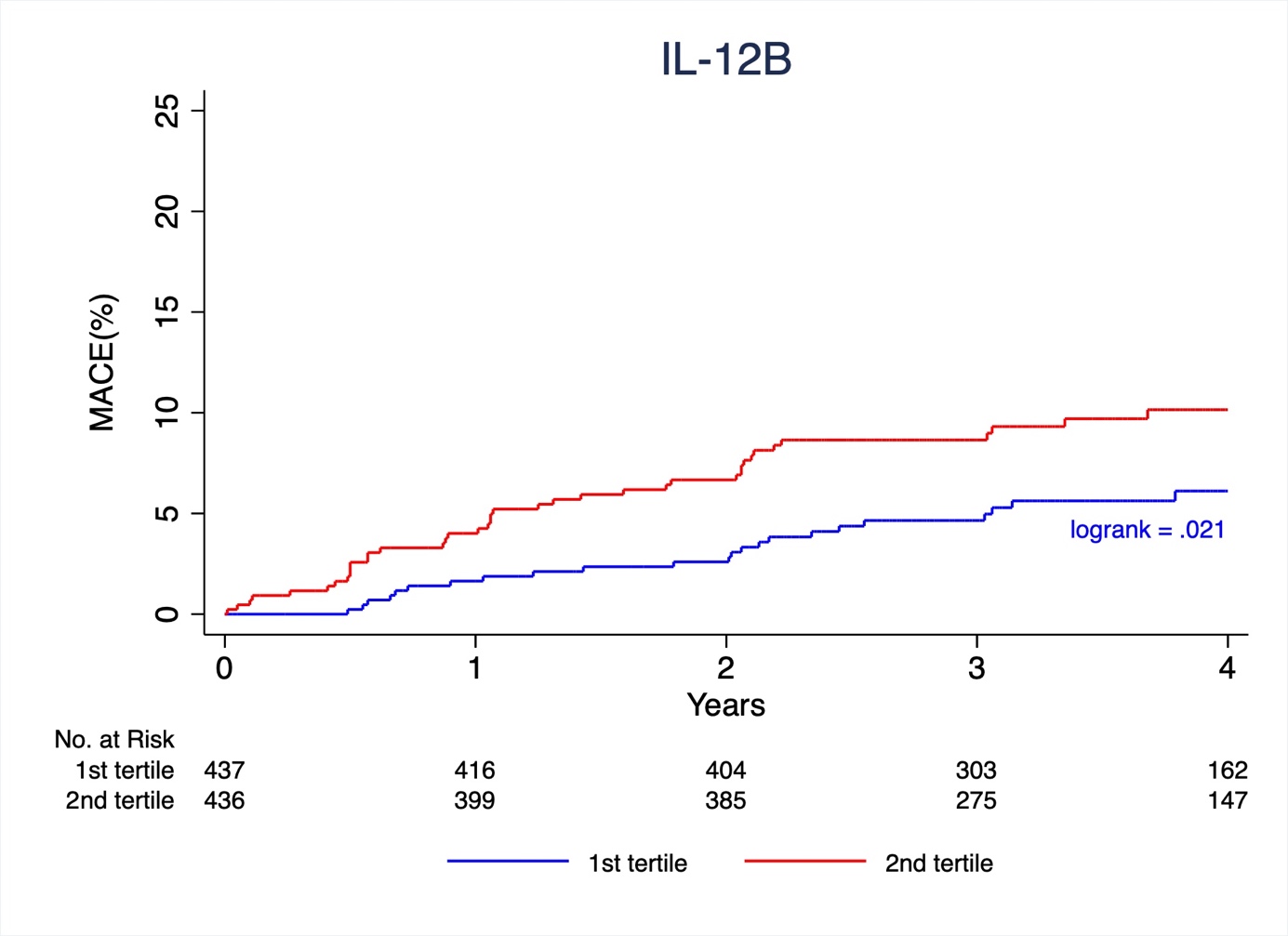
**

**Figure S1 G
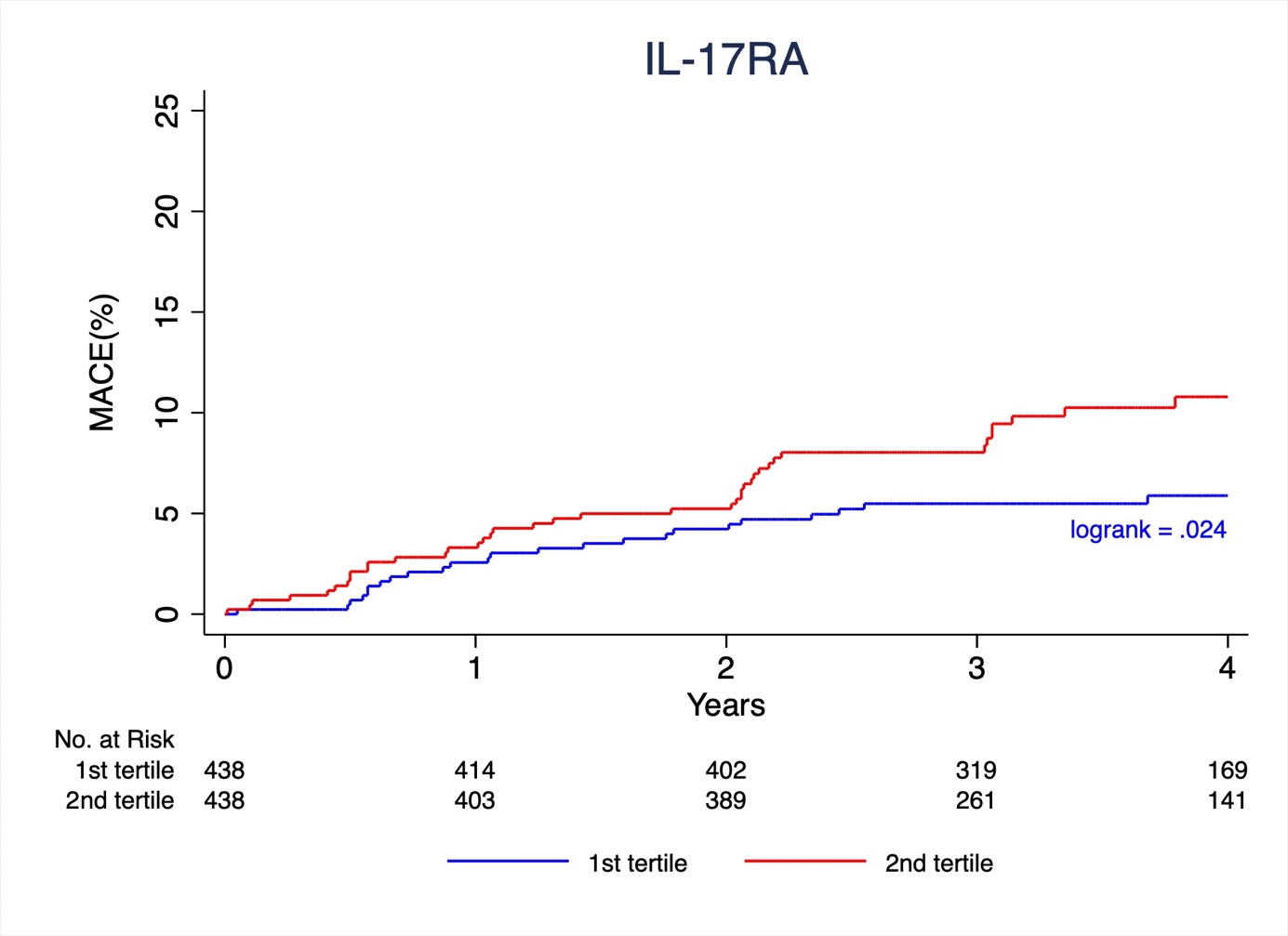
**

**Figure S1 H**

**
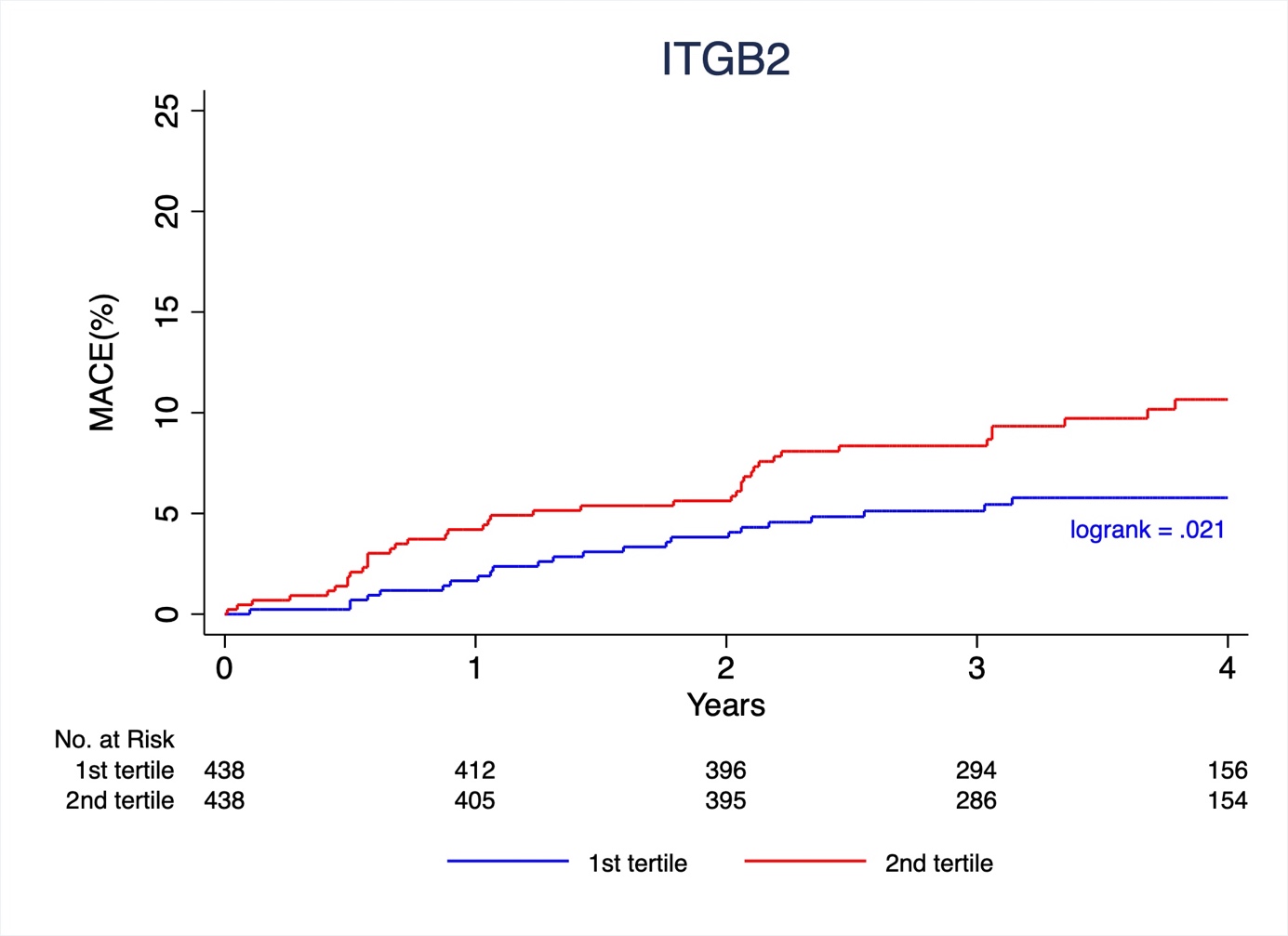
**

**Figuure S1 I**

**
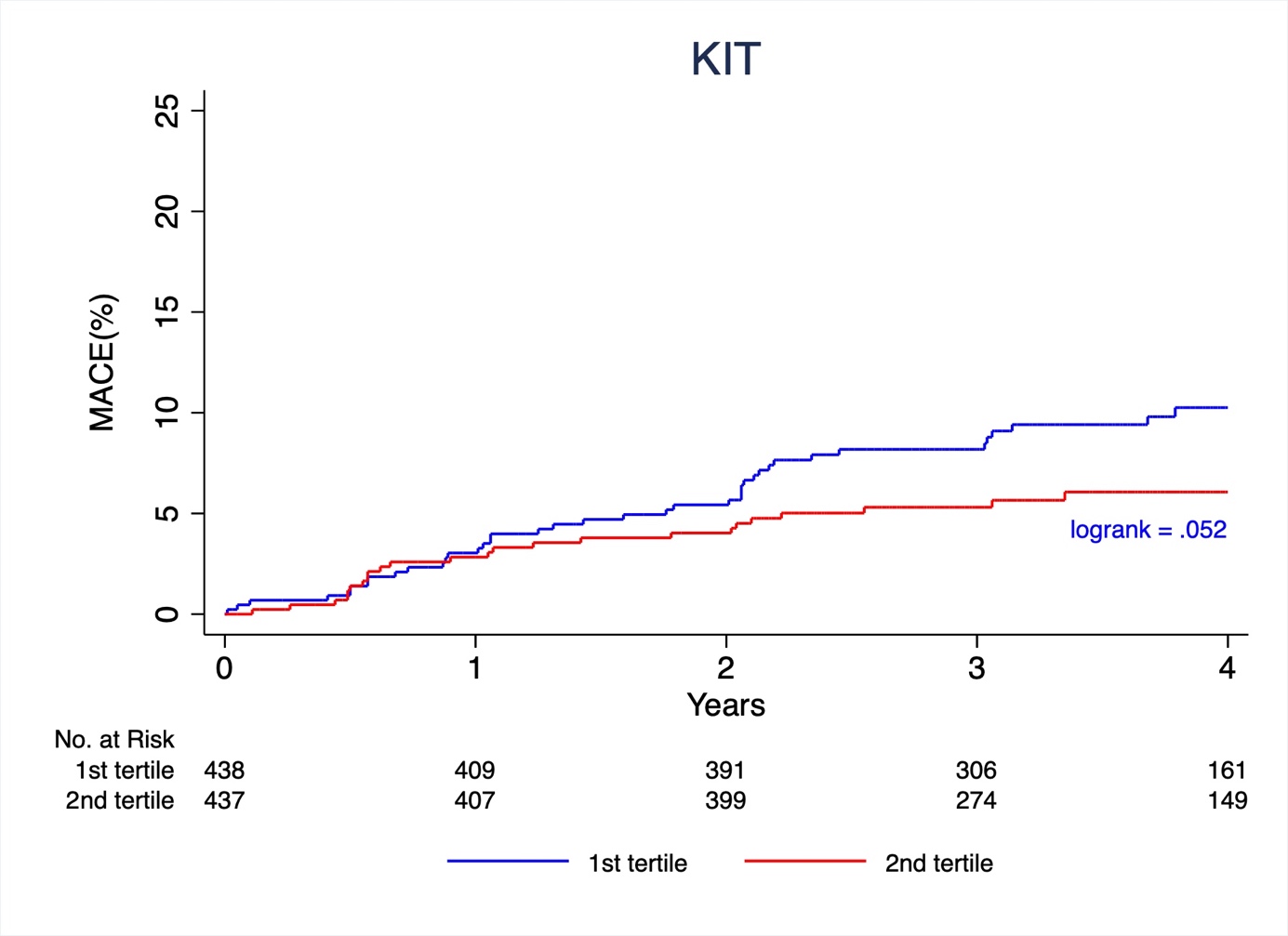
**

**Figure S1 J**

**
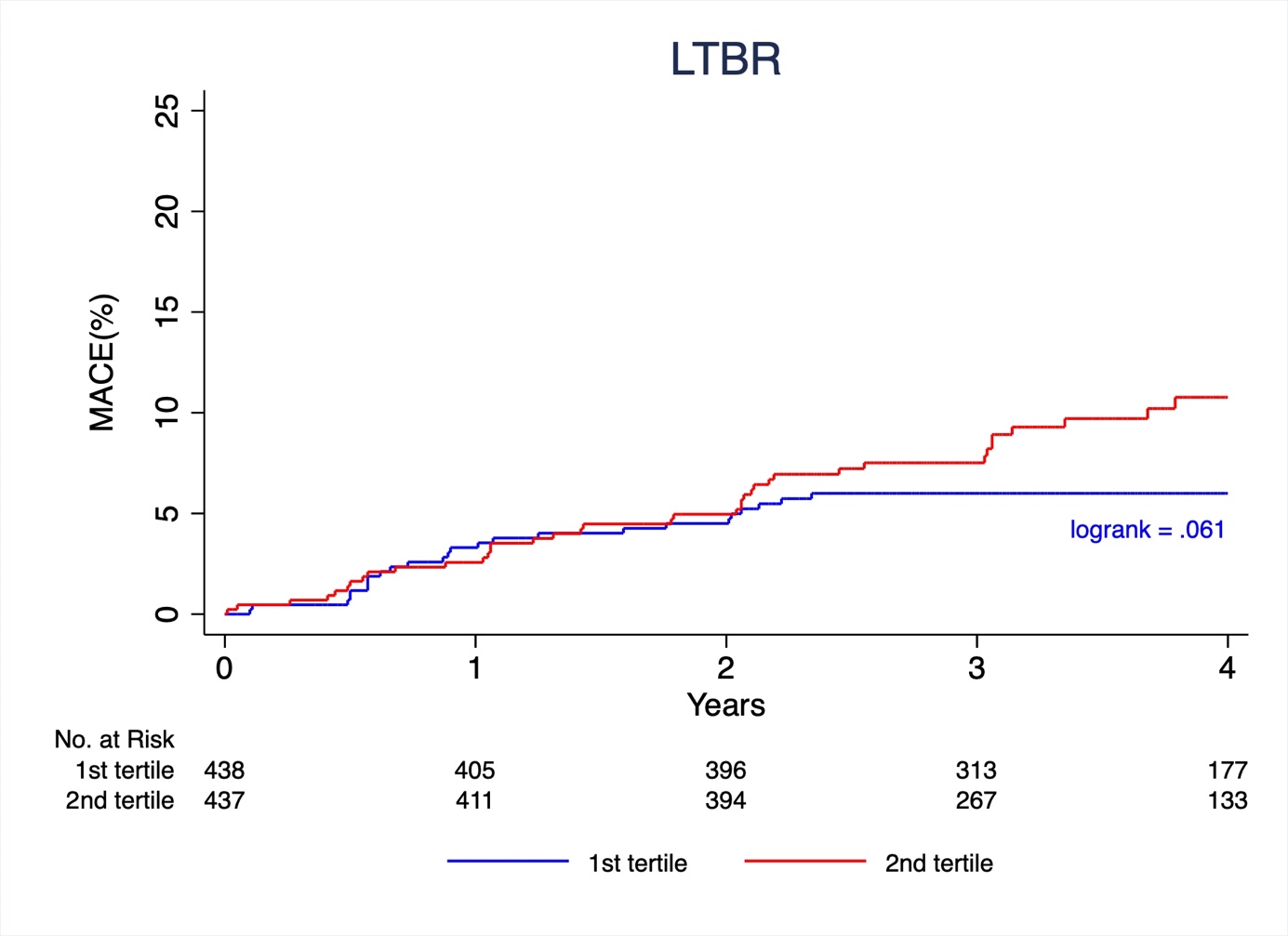
**

**Figure S1 K**

**
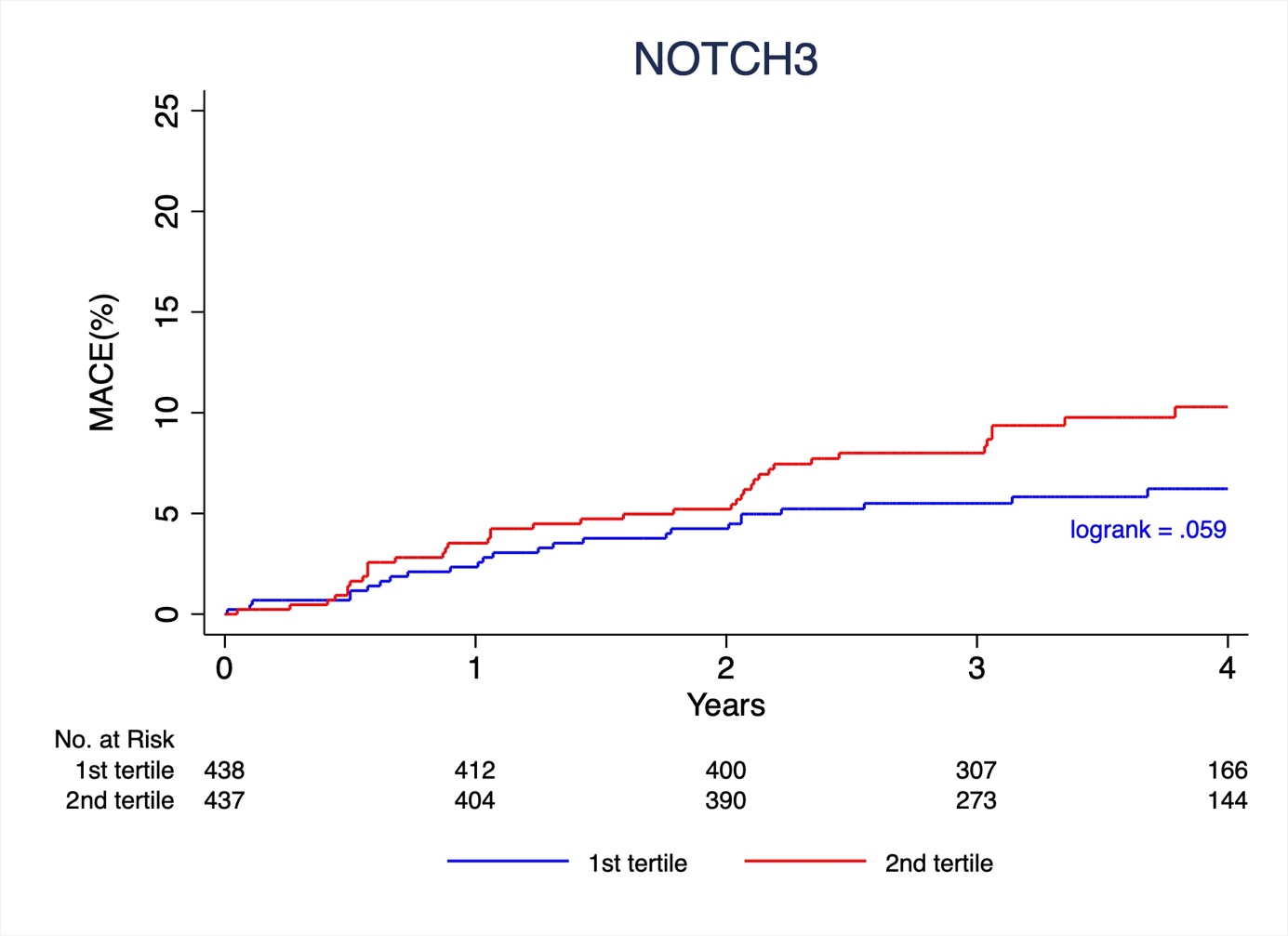
**

**Figure S1 L**

**
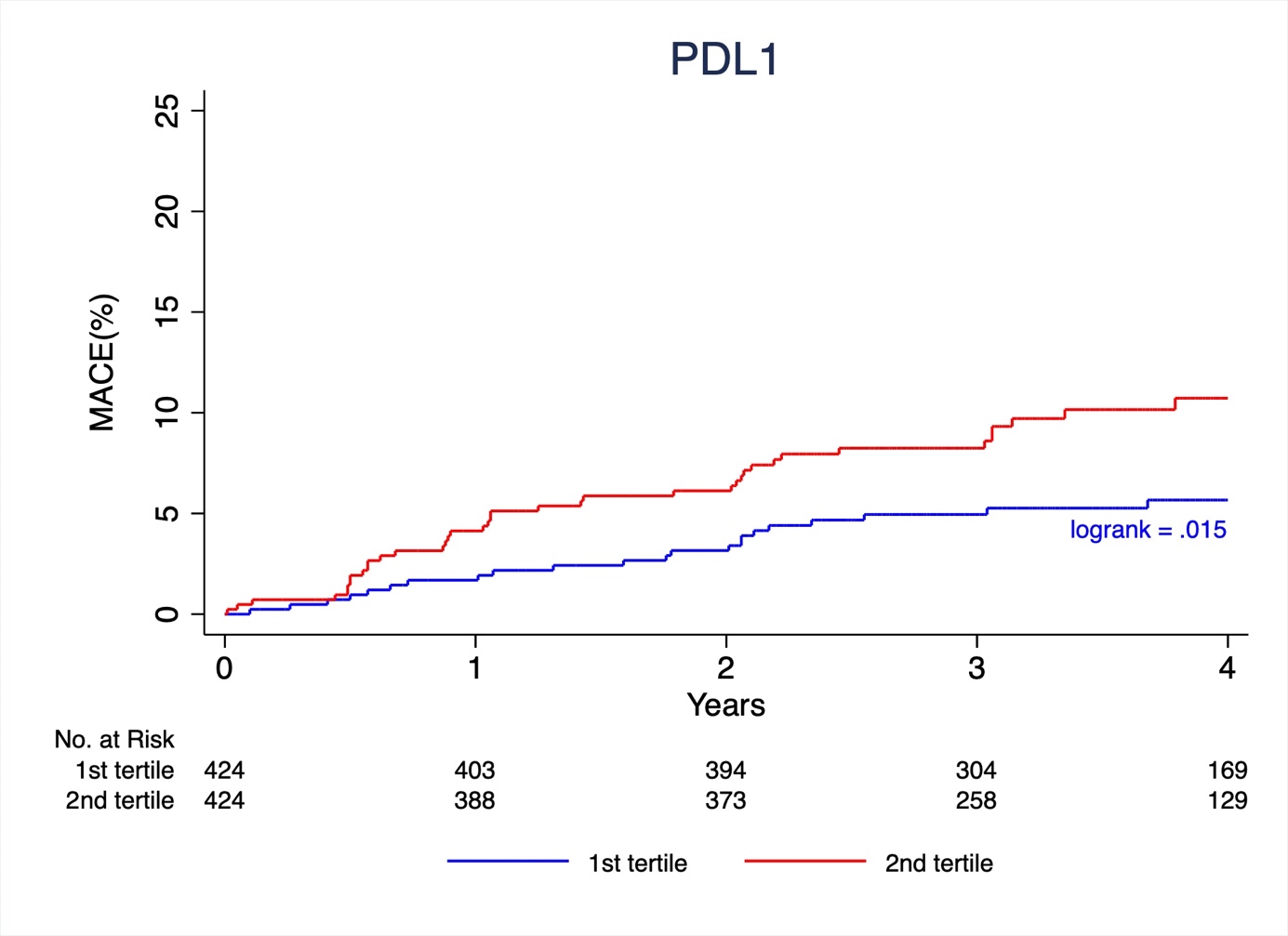
**

**Figure S1 M**

**
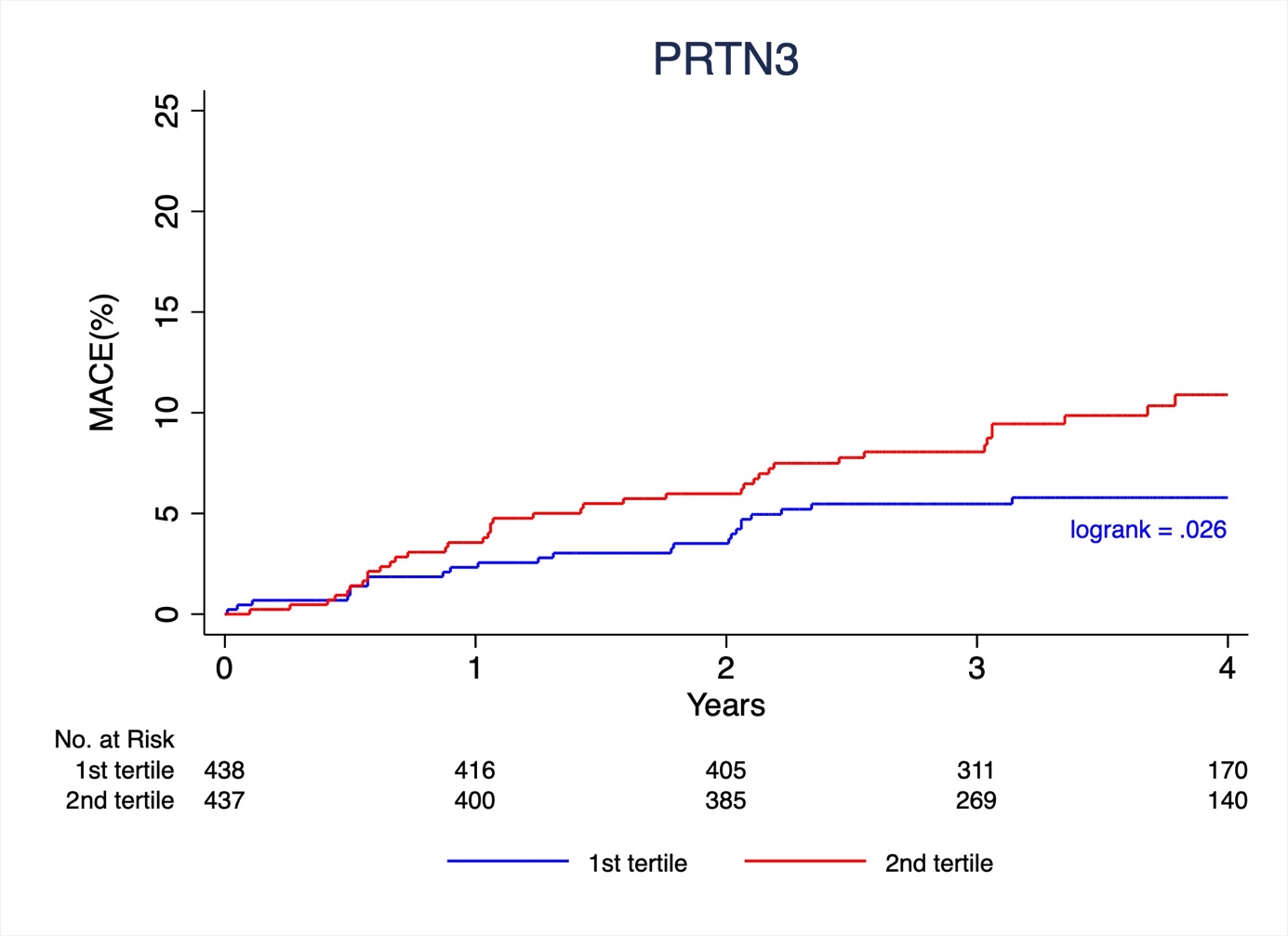
**

**Figure S1 N**

**
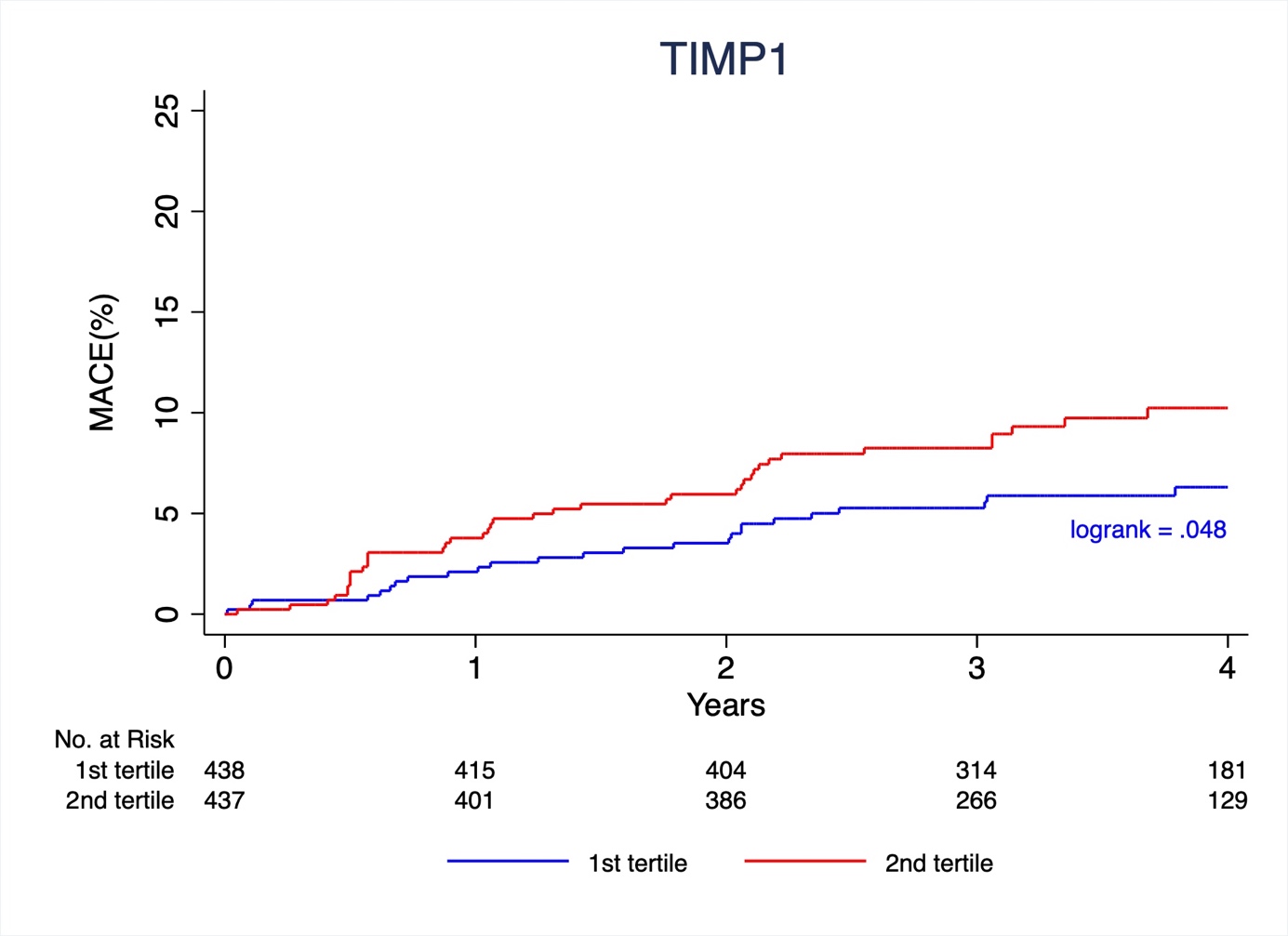
**

**Figure S1 O**

**
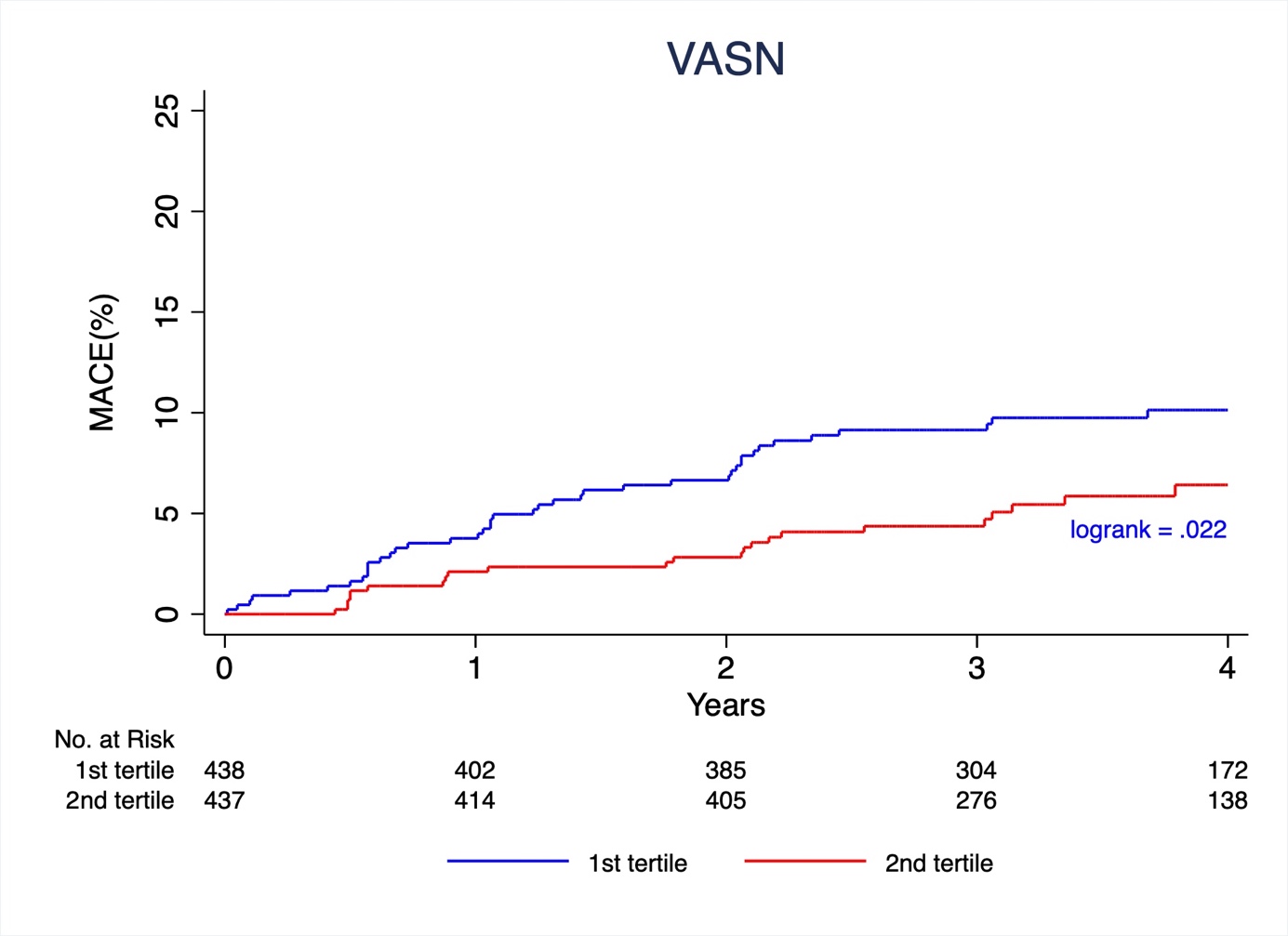
**


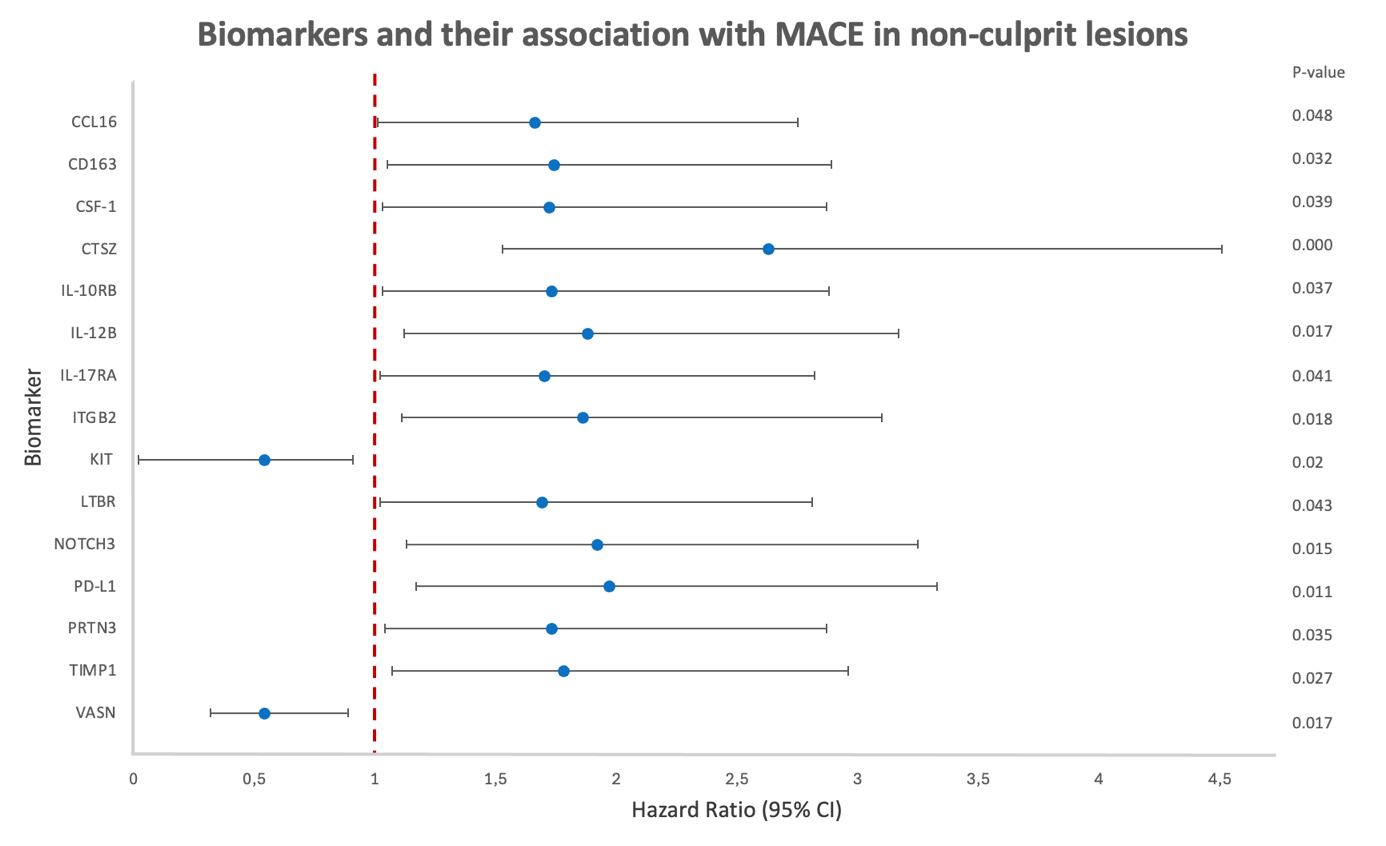


**Figure S2.** Forest plot presenting 7 biomarkers with individual, significant associations to MACE in non-culprit lesions in patients with recent MI. Results are presented as hazard ratio’s divided into two groups by the median with a 95% confidence interval. Models are adjusted for age and sex. HR indicates hazard ratio; CI, confidence interval; *P*, P value.
